# Feature-Based Molecular Network-Guided Dereplication of Natural Bioactive Products from Leaves of *Stryphnodendron pulcherrimum* (Willd.) Hochr

**DOI:** 10.3390/metabo11050281

**Published:** 2021-04-29

**Authors:** Paulo Gomes, Luis Quirós-Guerrero, Consuelo Silva, Sônia Pamplona, Jean A. Boutin, Marcos Eberlin, Jean-Luc Wolfender, Milton Silva

**Affiliations:** 1 Laboratory of Liquid Chromatography, Institute of Exact and Natural Sciences, Federal University of Pará, Belem 66075-110, Brazil; wendergomes@ufpa.br (P.G.); yumikoyoshioka@yahoo.com.br (C.S.); sgpamplona@yahoo.com.br (S.P.); 2 School of Pharmaceutical Sciences, University de Geneva, CMU, Rue Michel-Servet 1, CH-1206 Geneva, Switzerland; Luis.Guerrero@unige.ch (L.Q.-G.); Jean-Luc.Wolfender@unige.ch (J.-L.W.); 3 Institute of Pharmaceutical Sciences of Western Switzerland, University of Geneva, CMU, Rue Michel Servet 1, 1211 Geneva, Switzerland; 4 Pharmaceutical Science Post-Graduation Program, Faculty of Pharmacy, Federal University of Pará, Pará 66075-110, Brazil; 5 Institut de Recherches Internationales Servier, 50 rue Carnot, 92284 Suresnes, France; ja.boutin.pro@gmail.com; 6 MackMass Laboratory, School of Engineering—PPGEMN, Mackenzie Presbyterian University, São Paulo 01302-907, Brazil; marcos.eberlin@mackenzie.br

**Keywords:** *Stryphnodendron*, UHPLC-MS/MS, molecular network, dereplication

## Abstract

*Stryphnodendron pulcherrimum* is a species known to have a high content of tannins. Accordingly, its preparations are used in southern Pará, Brazil, for their anti-inflammatory and antimicrobial activities, but so far, its chemical profile composition remains essentially unknown. We herein describe the compounds present in a hydro-acetonic extract from *S. pulcherrimum* leaves as revealed by dereplication via ultra-high performance liquid chromatography coupled to high-resolution mass spectrometry. The data were combined with spectral organization, spectral matching through the Global Natural Products Social platform, in silico annotation and taxonomical ponderation. Several types of phenolic compounds were identified such as gallic acids, flavan-3-ols and flavone-like compounds. From these, 5 have been recently reported by our group, whereas 44 are reported here for the first time in this tree species, and 41 (out of 49) for this genus. The results highlight the possible role of *Stryphnodendron pulcherrimum* as a renewable source for natural bioactive products with potential pharmaceutical applications.

## 1. Introduction

*Stryphnodendron pulcherrimum* (Wild.) Hochr species belong to the Fabaceae family and are usually found in the Amazon and Atlantic forests and in the southern part of Bahia state in Brazil. This tree is commonly known in Brazil as “fake-barbatimão”, paricazinho, paricarana, jubarbatimão, juerana-branca or cowboy [1]. In southern Pará, its leaves, fruits and bark are used in traditional medicine, being particularly indicated as an anti-inflammatory agent [2]. A recent study [3] of several plant extracts from the Amazon region highlighted the antimicrobial activity of the aerial parts of *S. pulcherrimum* against strains of *Enterococcus faecalis*, proposing this plant as a potential source of natural bioactive compounds.

The use of natural products as drugs or as inspiration to develop new active principles currently plays an important role in the pharmaceutical industry [4]. This role has motivated researchers aiming to develop new strategies to study plant metabolites in a more comprehensive and rational manner. Plants offer vast, renewable and sustainable sources of new pharmacophores with chemical structures exquisitely designed for biological functions, and many of these are yet to be discovered [4,5,6].

The comprehensive chemical description of complex natural mixtures remains as an extremely challenging task [7]. Due to high composition complexity, they often contain several hundreds of compounds of contrasting properties [8,9]. This task has been, however, facilitated thanks to the continuous improvements in analytical techniques such as nuclear magnetic resonance (NMR) spectroscopy and liquid chromatography coupled to tandem mass spectrometry (LC-MS/MS) [10,11,12]. Recent developments of innovative strategies to study metabolites from complex matrices such as the organization of the spectral information using Global Natural Product Social Molecular Networking (GNPS)have also been very useful. This latter methodology is capable of simultaneously visualizing the chemical space from non-targeted mass spectrometry data and identifying compounds through mass spectral matching [13].

Efforts to identify metabolites of *Stryphnodendron* species have been intense and increasing due to the promising biological activities reported [3,14,15,16,17,18,19,20,21,22,23,24,25,26,27,28,29]. Tannins have been found to be their major components, particularly in the “barbatimao” species, and are likely responsible for the reported antimicrobial activities [30,31].

Herein, in the search for bioactive compounds, we used UHPLC-MS/MS combined with feature-based molecular networking-Global Natural Products Social (FBMN-GNPS) and other state-of-the-art bioinformatic tools to further decipher the chemical composition of the hydro-acetonic leaf extract of *Stryphnodendron pulcherrimum*.

## 2. Results

### 2.1. Stryphnodendron Pulcherrimum Extract Characterization by UHPLC-MS/MS

**Figure 1:**
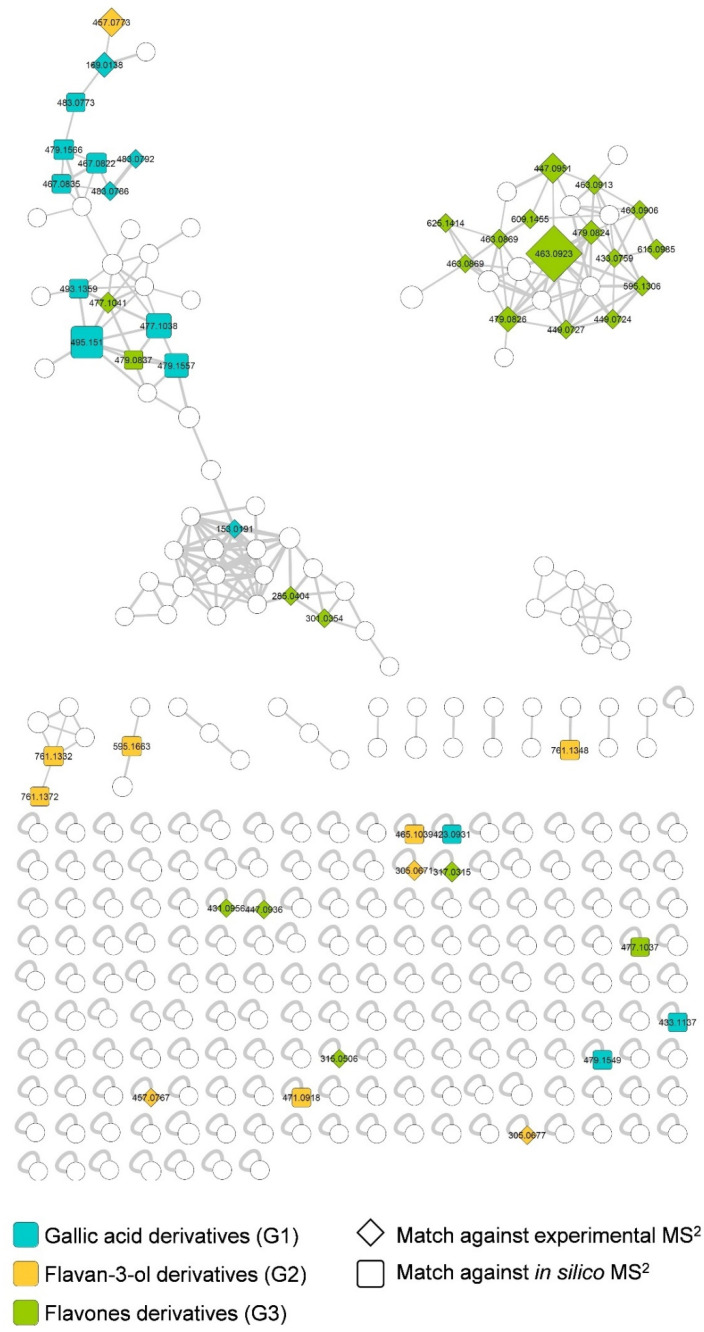
Molecular network from UHPLC-MS/MS data in the negative ion mode for *S. pulcherrimum* leaf extract. Numbers inside the nodes correspond to the *m*/*z* accurate masses (*Da*) for the [M–H]^−^ for each precursor ion. Node size is proportional to the intensity of the ion peaks in the total ion chromatogram. Node colors represent the three different groups: gallic acid derivatives (G1, blue), flavan-3-ol derivatives (G2, orange) and flavone derivatives (G3, green). Rhomboid shapes correspond to a match against an experimental MS^2^; square shapes are identifications from in silico spectra matching.

The hydro-acetonic extract was cleaned up using a C-18 SPE cartridge to remove highly lipophilic compounds such as chlorophylls. It was subjected to UHPLC-MS/MS in the negative ion (NI) mode to favor the ionization of phenolic compounds known to be abundant in plants from the genus. This high-resolution MS analysis using a Qtof analyzer provided nearly accurate molecular masses (MS^1^) and the corresponding fragmentation patterns (MS^2^) of the molecular ions [32]. To obtain a molecular network, the MS data were treated using MZmine software [33] and then, the data MS^1^ and MS^2^ were uploaded to the GNPS platform [34]. The molecular network (MN) obtained consisted in 284 features (Figure 1). The resulting MN was then subjected to dereplication against experimental MS^2^ data from the GNPS databases, and in silico fragmentation spectra created from a large database of natural products, followed by a re-ranking of the putative identities based on taxonomy [35,36].

According to the dictionary of natural products (DNP, v.29.1,), 42 compounds have been reported in the genus *Stryphnodendron*. From them, 36 correspond to saponin derivatives, mostly stryphnosides, which have been reported in fruits from *Stryphnodendron fissuratum* [37,38] and *Stryphnodendron coriaceum* [39]. The remaining six compounds include polyphenolic derivatives from *Stryphnodendron adstringens* [14,15,40]. Since the working sample is from leaves, not the fruits, an extensive literature research was conducted to create an in-house database (Table S1). It included 74 compounds reported for the *Stryphnodendron* genus, in different parts of the plants [14,15,16,17,23,24,29,40,41,42,43,44,45,46,47]. Based on the molecular formula (MF) and heuristic filtering [48], this database was used to further identify compounds in the extract.

**Figure 2:**
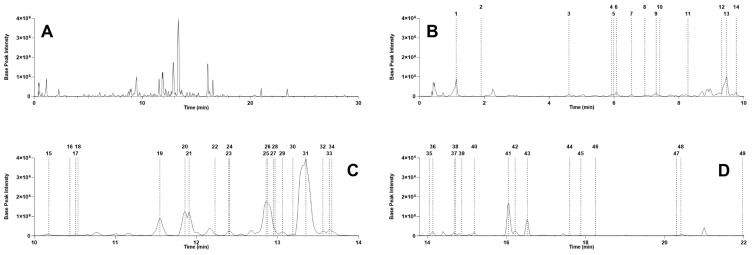
(**A**) UHPLC-MS/MS total ion chromatograms in the negative ion mode for the leaf extract of *Stryphnodendron pulcherrimum*. Zoom sections of the chromatograms for (**B**) 0 to 10 min, (**C**) 10 to 14 min and (**D**) 14 to 22 min. Dashed lines represent the position (retention time) of each dereplicated compound.

After the dereplication process and careful evaluation of the proposed annotations, 49 compounds (**1–49**) were putatively identified (Table 1 and Figure 2). They were all found to be phenolics. These compounds were subdivided into three groups based on their structural characteristics, independent of their retention times, namely: Group 1 of gallic acid derivatives (**1**, **2**, **8–11**, **14**, **15**, **21**, **25**, **26**, **32**, **38**, **43**, **44**), Group 2 of flavan-3-ol derivatives (**3**, **4**, **6**, **7**, **12**, **13**, **16**, **18**, **22**, **24**) and Group 3 of flavone derivatives (**5**, **17**, **19**, **20**, **23**, **27–31**, **33–37**, **39–42**, **45–49**). Gallic acid (**1**), gallocatechin (**4**), (epi)gallocatechin (**6**), (epi)gallocatechin gallate (**13**), 3-*O*-galloyl-4′-*O*-methylepigallocatechin (**24**), myricitrin (**31**), myricetin (**42**) and luteolin-4′-*O*-glucoside (**45**) have been previously reported in the genus *Stryphnodendron* [14,15,17,23,24,29,41,42,43,44,45,46,49]. Among them, gallic acid (**1**), (epi)gallocatechin (**6**), (epi)gallocatechin gallate (**13**), myricitrin (**31**) and myricetin (**42**) have been reported in *S. pulcherrimum* [49]. To the best of our knowledge, 44 of these compounds are reported herein for the first time for *S. pulcherrimum* and 41 for the genus.

### 2.2. Structural Identification of Compounds Present in the Extract of S. pulcherrimum

**Figure 3:**
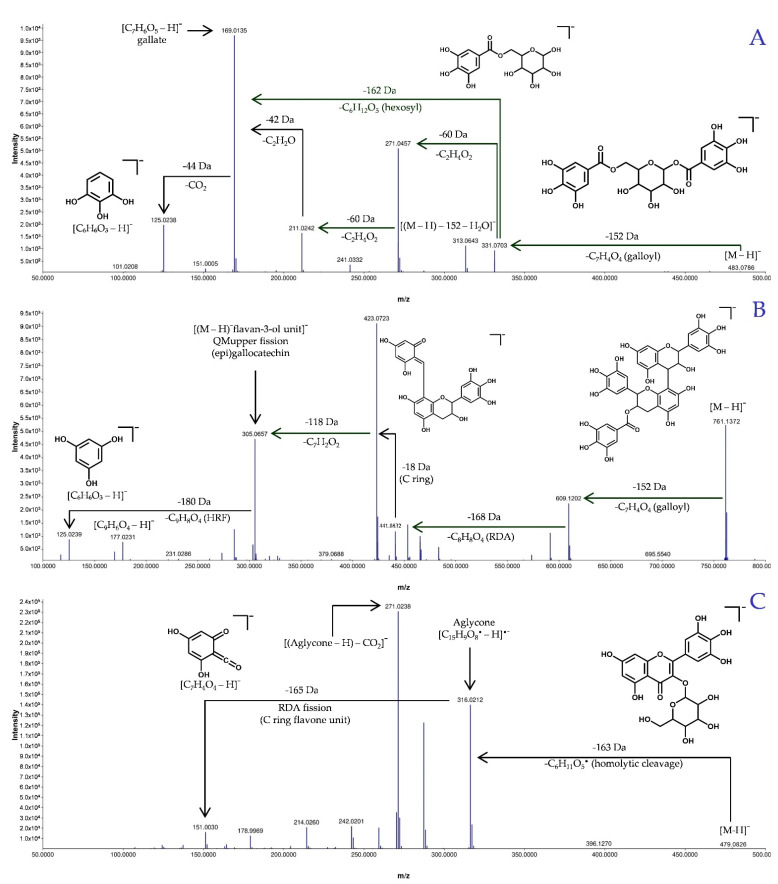
MS/MS for representative [M–H]^−^ molecular ions from each group. Group 1 (**A**), [3,4,5-trihydroxy-6-(3,4,5-trihydroxybenzoyl)oxyoxan-2-yl]methyl-3,4,5-trihydroxybenzoate (**9**), gallic acid derivatives; Group 2 (**B**), 3-*O*-galloyl-(epi)gallocatechin-(epi)gallocatechin (**22**), flavan-3-ol derivatives; and Group 3 (**C**), myricetin-3-galactoside (**20**), flavone derivatives.

**Figure 4:**
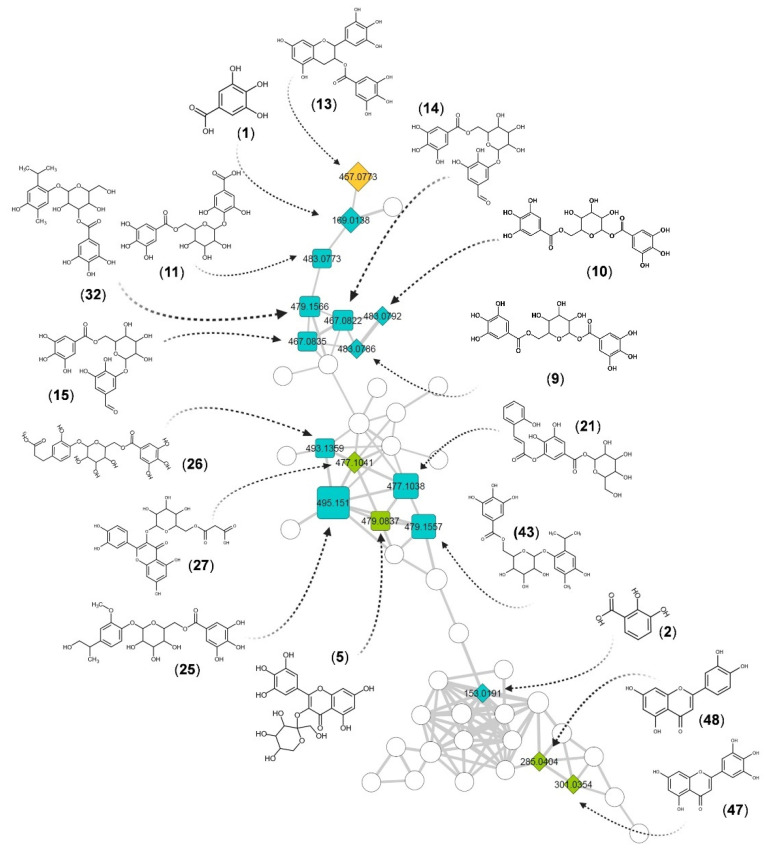
Gallic acid derivatives in the largest cluster of the network. Numbers inside the nodes correspond to the accurate mass for each precursor. Node size is proportional to the intensity of the ions in the total ion chromatogram. Node colors represent structural groups: gallic acid derivatives (G1, blue), flavan-3-ol derivatives (G2, orange) and flavone derivatives (G3, green). Rhomboid shapes correspond to a match against an experimental MS^2^; square shapes are identifications from in silico spectra matching.

**Figure 5:**
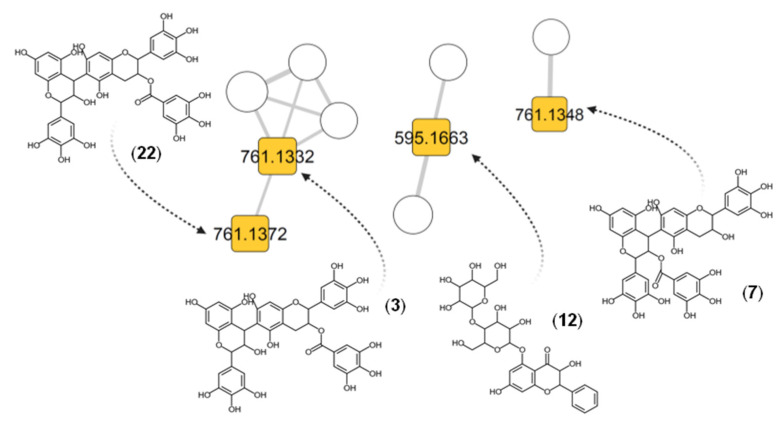
Illustrative compounds assigned as flavan-3-ol derivatives (Group 2). Numbers inside the nodes correspond to the accurate mass for each precursor. Node size is proportional to the intensity of the ion peaks in the total ion chromatograms. Node colors represent the three different groups: gallic acid derivatives (G1, blue), flavan-3-ol derivatives (G2, orange) and flavone derivatives (G3, green). Rhomboid shapes correspond to a match against an experimental MS^2^; square shapes are identifications from in silico spectra matching.

**Figure 6:**
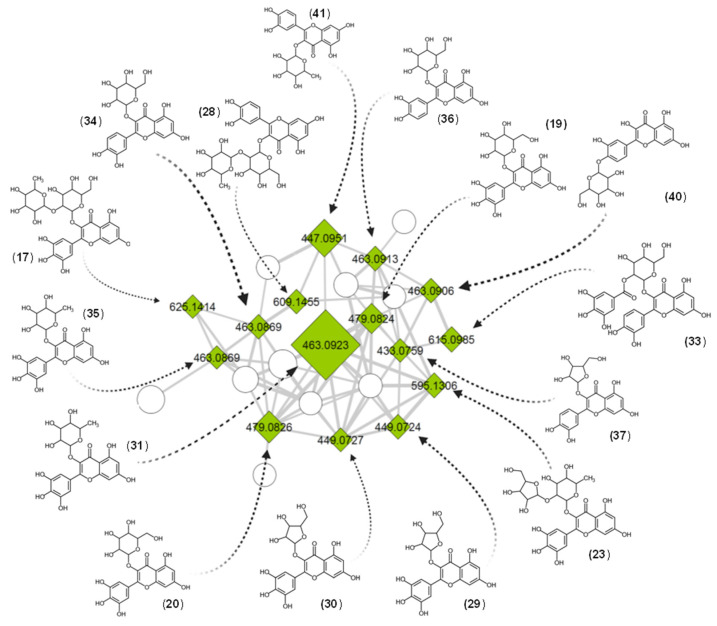
Compounds assigned mainly as flavone derivatives in the cluster belonging to Group 3. Numbers inside the nodes correspond to the accurate mass for each precursor. Node size is proportional to the intensity of the ions in the total ion chromatogram. Node colors represent the three different groups: gallic acid derivatives (G1, blue), flavan-3-ol derivatives (G2, orange) and flavone derivatives (G3, green). Rhomboid shapes correspond to a match against an experimental MS^2^; square shapes are identifications from in silico spectra matching.

According to our annotation results, 30 out 49 compounds had a spectral match against the experimental database from GNPS, while the remaining 19 had a match against the in silico database. To corroborate the structural proposals for these 19 features, their individual fragmentation patterns were explained. Figure 3 display examples of fragmentation patterns for each structural group. Group 1 includes conjugates of gallic acid with sugars and small organic acids (Figure 4). Fragmentation patterns of these compounds are characterized by the ions of *m*/*z* 169 (gallate) and 125 (3,4,5-trihydroxybenzene, ion derived from the decarboxylation of the gallate) [50,51]. If a sugar unit is present, common losses of H_2_O and consecutive losses of C_2_H_4_O_2_ units from the residual hexose and the heterolytic cleavage of the sugar are observed [52]. For Group 2 (flavan-3-ol derivatives, Figure 5), the most characteristic fragmentation includes quinone methide fission (QM), heterolytic ring fission (HRF) and retro-Diels–Alder (RDA) cleavage, which inform the hydroxylation patterns in the different rings (A, B and C) [53,54] and the identity of the monomeric units. Group 3 is mainly composed of glycosylated flavones (Figure 6). Their fragmentation usually yields a fragment ion of *m*/*z* 316 [C_15_H_9_O_8_^•^-H]^•−^, which points to homolytic cleavage and loss of the glycoside radical (sugar unit) [55,56]. Fragment ions of *m*/*z* 301 and 300 are both indicative of heterocyclic or homolytic cleavage and loss of the glycoside [56]. The fragment ion [(Aglycone-H)-CO_2_]^−^ of *m*/*z* 271 is also usually formed. The relatively light fragment ion of *m*/*z* 151 is produced by RDA cleavage of the aglycone [54,57].

The gallic acid derivative group, contributing to the main part of the cluster of Figure 4, covers a wide range of polarities (RT from 1 to 17 min, Figure 2). For example, **8** was putatively assigned to 6-*O*-galloylarbutin. Its fragment ion of *m*/*z* 313 is likely produced by loss of dihydroxybenzene [M–H–C_6_H_6_O_2_]^–^, whereas that of *m*/*z* 253 is likely from loss of the gallate moiety [M–H–C_7_H_6_O_5_]^–^. The fragments ions of *m*/*z* 169 and 125 seem to be formed after a gallate loss followed by consecutive losses of CO_2_. As another example, **11** was assigned as gallic acid *O*-galloyl glucoside, an isomer of **9** and **10**. For this isomer, we proposed that the C–O–C bridge between the hexose and a gallic acid moiety is linked at C4 of the benzoate. In such connectivity, this bond is weaker, which could explain the variation in abundance of fragment ions with respect to **9** and **10**. The fragment ion of *m*/*z* 169 [M–H–C_13_H_16_O_9_]^−^ is the base peak, suggesting cleavage of the C–O bond (C2 in the hexose) and loss of the hexose and gallate groups as neutral species. CO_2_ loss likely forms the fragment ion of *m*/*z* 125 [M–H–C_13_H_16_O_9–_CO_2_]^−^.

We identified 10 compounds, as illustrated in Figure 5, in the flavan-3-ols category, presenting a 2-phenyl-3,4-dihydro-2H-chromen-3-yl backbone [58]. These types of compounds are commonly used as functional/nutritional agents for beverages, fruits and vegetables, food grains, herbal remedies, dietary supplements and dairy products [59], or as ligand for protein inactivation, which prevent the growth of microorganisms [31].

For example, three C_37_H_30_O_18_ isomers: [M–H]^−^ of *m*/*z* 761.1332 (**3**, 4.61 min), 761.1348 (**7**, 6.54 min) and 761.1372 (**22**, 12.23 min) were assigned as (epi)gallocatechin dimmer conjugates: 3-*O*-galloyl-(epi)gallocatechin-(epi)gallocatechin (**3**)**,** (epi)gallocatechin-3′-*O*-galloyl-(epi)gallocatechin (**7**) and 3-*O*-galloyl-(epi)gallocatechin-(epi)gallocatechin. The MS^2^ of **22** is illustrated in Figure 3B. These three isomeric [M–H]^−^ yielded common fragment ions such as *m*/*z* 609 for the loss of the gallate group [M–H–C_7_H_4_O_4_]^−^, *m*/*z* 591 for the loss of H_2_O from the C ring [M–H–C_7_H_4_O_4_–H_2_O]^−^), *m/z* 441 for RDA [M–H–C_7_H_4_O_4_–C_8_H_8_O_4_]^−^, *m*/*z* 423 for the loss of H_2_O [M–H–C_7_H_4_O_4_–C_8_H_8_O_4_–H_2_O]^−^), *m*/*z* 305 from the loss of a monomeric unit (quinone methide reaction [60]) and *m/z* 125 from further loss of a C_9_H_8_O_4_ unit. Fragment ions of *m*/*z* 593 and 575 (**7** and **22**) likely result from further consecutive losses of H_2_O [M–H–C_7_H_4_O_4_–2H_2_O]^−^. The fragment ion of *m/z* 405 likely results from RDA followed by double H_2_O losses [M–H–C_8_H_8_O_4_–2H_2_O]^–^. The ion of *m*/*z* 287 [M–H–QM–H_2_O]^–^ likely results from the monomeric unit of *m*/*z* 305. Further HRF reactions seem to produce the ion of *m*/*z* 243 [M–H–QM–C_2_H_6_O_2_]^–^, and the ion of *m*/*z* 169 seems to correspond to the gallate anion. Compounds **3** and **22** showed additional fragment ions of *m*/*z* 483, 465 and 177. For [M–H–C_7_H_4_O_4_–C_6_H_6_O_3_]^−^ of *m*/*z* 483, loss of 1,3,5-trihydroxybenzene seems likely, followed by H_2_O loss [M–H–C_7_H_4_O_4–_C_6_H_6_O_3–_H_2_O]^−^ to yield the ion of *m*/*z* 465. The fragment ion of *m*/*z* 177 has been reported [60] and seems to result from the ion of *m*/*z* 305 by C_6_H_8_O_3_ loss, but the corresponding pathway is still unknown. However, **3** showed unique fragment ions of *m*/*z* 573, 453, 355 and 285. That of *m*/*z* 573 seems to result from *m*/*z* 591 by H_2_O loss [M–H–C_7_H_4_O_4_–2H_2_O]^−^, whereas subsequent C_7_H_4_O_2_ loss yields *m*/*z* 453. The ion of *m*/*z* 355 has a [M–H–338–C_3_O_2_]^−^ composition and likely results from that of *m*/*z* 423 [61]. Compound **7** yielded fragment ions of *m*/*z* 717 [M–H–CO_2_]^−^ and *m*/*z* 635 [M–H–C_6_H_6_O_2_]^−^, both corresponding to trihydroxybenzene loss from [M–H]^–^ of *m*/*z* 761. We suggest that these ions point to the galloyl group position of this compound at C-3 on the extension unit of the flavone. Unlike compounds **3** and **22**, which differ in position (C-3 in the base unit of the flavone), probably conferring the weakest stability bond.

For the last group of chemical structures, a total of 24 flavone derivatives were detected and annotated (Figure 6). Flavones have a double bond between C2 and C3 (C ring) in the flavonoid structure and they are oxidized at C4 and are reported to have a variety of biological activities [62,63].

Compound **5** was assigned to myricetin 3-sorboside, based on its fragmentation pattern. From dereplication on ISDB, we suggest that its CH_2_OH group is not at the C-5 position of the hexose, as in myricetin 3-galactoside, but at C-1. This assignment is justified by the uncommon fragment ions of *m*/*z* 250 and 121. This structural difference may explain why **5** clustered with the gallic acid derivatives (Figure 1 and Figure S1). Compound **39** was assigned from ISDB data yielding fragment ions of *m*/*z* 405 ([M–H–C_3_H_4_O_2_]^–^ from ^3,5^X_1_ fragmentation on a glucopyranoside, *m*/*z* 316 from loss of a glucopyranoside [M–C_6_H_10_O_5_]^–•^) and *m*/*z* 300 from CH_3_^•^ loss [M–C_6_H_10_O_5_–CH_3_]^–•^, as 3′,5,7,8-tetrahydroxy-4′-methoxyflavone-8-*O-*glucopyranoside. For more detailed descriptions of the remaining dereplicated ions, refer to the Supplementary information. The fragmentation pathways for the major compounds (**13**, **21**, **25**, **31** and **41**) are presented in Figures S4–S8.

## 3. Discussion

Several *Stryphnodendron* species have been the focus of chemical studies to identify their chemical composition, and to assess the natural products responsible for the reported biological activities, such as anti-inflammatory and antimicrobial [3,14,15,16,17,18,19,20,21,22,23,24,25,26,27,28,29]. The many uses of these plants in Brazilian folk medicine point to them as possible sources of bioactive compounds [30,31].

In this study, we aimed to comprehensively describe the composition of the leaves *Stryphnodendron pulcherrimum* based on the annotation of all metabolites detected in the hydro-acetonic extract by UHPLC-MS/MS. The combination of spectral organization through MN [34] and the search against both experimental and in silico spectral databases considerably reduces the time and resources for the putative identification of a considerable number of metabolites. Combining the MN with matching of MS/MS against in silico databases [35] was possible re-weighting of the putative candidates based on the biological source and taxonomy [36]. Also, filtering with MF assignment and manually verifying major fragmentation patterns allowed us to significantly increase the confidence of the metabolite annotation.

All compounds reported here, mainly small phenolic conjugates and polyphenols, derive either from the upper stream of the shikimate, phenylpropanoid or phenylpropanoid-acetate pathway. The MN presents two major clusters. The largest one is mainly constituted by compounds from G1 (Figure 4) which are derivatives and conjugates of gallic acid (**1**). Note that gallic acid displays several biological activities and is considered a biomarker for the chemotaxonomy and differentiation of *Stryphnodendron* species because of its recurrence and concentration [43,64,65,66,67].

The second G3 major cluster, composed entirely of flavone derivatives (Figure 6), contains the most abundant compounds according to their ion intensities in the TIC trace (Figure 2). Myricitrin (**31**) displays a wide range of biological activities and is remarkable as an anti-inflammatory and antioxidant agent [68,69,70,71,72,73]. Additionally, a smaller group of compounds bearing a flavan-3-ol core structure was putatively identified. We unequivocally detected and identified gallocatechin and (epi)gallocatechin (**4**, **6**). These, along with catechin and other monomeric units, are the precursors of most condensed tannins [59,74]. The added value of this type of study lies in previous findings in which some of the identified compounds have been reported in other biological sources as promising bioactive agents for the pharmaceutical industry.

Some examples of these well-characterized compounds include (epi)gallocatechin gallate (**13**), coumaroyl-*O*-galloyl-glucose (**21**), (3,4,5-trihydroxy-6-(4-(1-hydroxypropan-2-yl)-2-methoxyphenoxy)tetrahydro-2H-pyran-2-yl)methyl-3,4,5-trihydroxybenzoate (**25**), myricitrin (**31**) and quercitrin (**41**), which are the major compounds in the studied extract of *S. pucherrimum*. (Epi)gallocatechin gallate (**13**) and its analogues have been reported as proteasome inhibitors [75], preventers of degenerative brain diseases [76], antibacterial [77], antiviral, antifungal [78], part of cancer treatments [79,80] and modulators of cyclooxygenase-2, oxidative stress and inflammation of different cellular processes [80]. Coumaroyl-*O*-galloyl-glucose (**21**) has been reported in extracts with wound healing properties [81] and antioxidant, anti-inflammatory, antimutagenic, antimicrobial [82] and antidiarrheal activities [83]. Similarly, (3,4,5-trihydroxy-6-(4-(1-hydroxypropan-2-yl)-2-methoxyphenoxy)tetrahydro-2H-pyran-2-yl)methyl-3,4,5-trihydroxybenzoate (**25**) is a natural product present in traditional Chinese medicine [84].

Myricitrin (**31**) was the most intensity compound found in the extract. It was recently proposed to pre-treat liver ischemia/reperfusion injury [69], and it also presents antithrombotic [70], osteoarthritis [71], antioxidant, anti-inflammatory and antifibrotic [72] activities. Quercitrin (**41**) was recently described to reduce the infarct volume in stroke in mouses [85], to display inflammatory [86], antiproliferative and anti-apoptotic effects on lung cancer cells [87] and to have cytoprotective effects against free radicals [88].

The metabolites present in the leaves of *S. pulcherrimum* have therefore great biological potential, some of them already proven, and some yet to be discovered, making this plant a promising new, natural and renewable Amazonian source of bioactive molecules. The dereplication process employed proved to be efficient, producing valuable information about the chemical composition in a considerably reduced time compared to classical phytochemical studies. Finally, the description of the various structurally new secondary metabolites.

## 4. Materials and Methods

### 4.1. Chemicals and Reagents

Sodium hypochlorite p.a. was acquired from Dinâmica (Jaraguá do Sul, SC, Brazil). Ethyl alcohol (99%) was acquired from Êxodo científica (Sumaré, SP, Brazil). Acetone chromatographic grade and methanol LC-MS grade were acquired from Tedia (Fairfield, OH, USA). Formic acid was purchased from Merck (Darmstadt, Germany). Ultrapure water was produced by a Direct-Q 5 system (Millipore, Merck Darmstadt, Germany).

### 4.2. Plant Material Collection and Extraction

*Stryphnodendron pulcherrimum* leaves were collected in September 2019 in Vigia (PA, Brazil). It is associated with the research project registered in SISGEN under number A678D8C. An exsiccate (Voucher IAN 199608) was deposited in the herbarium of Embrapa Amazônia Oriental, Pará, Brazil. The leaves were washed with water and then disinfected with a sodium hypochlorite solution (NaOCl, 0.1%), followed by ethyl alcohol (70% *v*/*v*). Then, the leaves were dried in an air circulation oven at 45 °C until constant weight. The dried leaves were crushed in a Fritsch pulverisette 14 ball mill (Idar-Oberstein, RP, Germany.), obtaining 1.866 kg of powder with a 60–100 µm particle size.

A total of 20 g of powder was extracted with 100 mL of H_2_O/Acetone (3:7) in an ultrasound bath, Branson 2510 (Danbury, CT, USA), for 40 min at 25 °C as previously reported [28]. The liquid was vacuum filtered, and the solvent was reduced under vacuum in a rotary evaporator Büchi Syncore (Flawil, Switzerland). A total of 6 g of dry extract (HCOE) was obtained. The extract was kept at −18 °C in a freeze Indrel (Londrina, PR, Brazil) until analysis.

### 4.3. Sample Preparation for UHPLC-MS/MS

HCOE (1 mg) was solubilized in 1 mL of H_2_O/Methanol (2:8 *v*/*v*) and passed through a 50 mg C18 Solid Phase Extraction (SPE, Phenomenex, Torrance, CA, USA) cartridge, previously conditioned with 1 mL of methanol and 1 mL of water. The filtrate was dried, and the solid residue was dissolved in 1 mL of methanol, filtered through a 0.22 µm hydrophilic filter (Millipore, Merk, Darmstadt, Germany) and diluted to 100 µg/mL.

### 4.4. UHPLC-MS/MS

The analyses were performed on a UHPLC coupled to an ESI-QTof Xevo G2-S Tof mass spectrometer (Waters Corp., Milford, MA, USA) with an electrospray ionization (ESI) probe operating in the negative ion mode. The *m*/*z* mass was 100–1200, and Leucine-enkephalin was used as a LockSpray reference compound. Analysis by UHPLC was carried out on a BEH C18 (50 × 2.1 mm, 1.7 μm) Waters column. The column and autosampler were kept at 40 and 25 °C, respectively. Elution was performed with ultra-pure water (solvent A) and methanol acidified with 0.1% formic acid (solvent B). The gradient method was set as follows: 0–2 min, 2% B 2–10 min, 2–10% B; 10–20 min, 10–20% B; 20–25min, 20–30% B; 25–30min, 30–40% B. The flow rate was 250 μL/min. The total ion chromatogram was acquired using Masslynx V4.1 software (Waters Corp., Milford, MA, USA). The mass spectrometry parameters were set to the following: desolvation gas flow (N_2_) at 600 L/h and desolvation temperature at 150 °C, the cone gas flow (N_2_) at 50 L/h and the source temperature at 120 °C. The capillary and sampling cone voltages were adjusted to 1.0 kV and 40 V, respectively.

The data-dependent experiments (DDA, MS/MS) were performed on the five most abundant ions detected in full-scan MS (top 5 experiments per scan). The ion peaks were detected at +1 and +2 charge states with the inclusion of the 10 most intense ion peaks with 0.2 Da (*m*/*z*) charge state tolerance and 2 Da extraction tolerance. The differentiation of molecular ions, adducts and fragment ions was conducted by chromatographic deconvolution with 3 Da de-isotope tolerance and 6 de-isotope extraction tolerance. The MS/MS isolation window width was 1 Da, and the stepped normalized collision energy (NCE) was set to 10, 20, 30, 40 and 50 eV units.

### 4.5. Mass Spectrometry Data Treatment Parameters

The UHPLC-MS/MS data were converted from RAW (Waters Corp., Milford, MA, USA) standard data format to mzXML format using MSConvert 3.0.2 [89]. The resulting file was treated using MZmine v2.53 [33]. For mass detection, at MS^1^ and MS^2^ levels, noise levels of 5.0E^2^ and 0.0E0 were used. The ADAP chromatogram builder algorithm was used and set to a minimum group size of scans of 3, minimum group intensity threshold of 5.0E^2^ and minimum highest intensity of 5.0E^2^ with an *m*/*z* tolerance of 12.0 ppm. The ADAP algorithm (wavelets) was used for chromatogram deconvolution. The intensity window S/N was used as S/N estimator with a signal to noise ratio set to 5, a minimum feature height of 5.0E^2^, a coefficient area threshold of 50, a peak duration range from 0.01 to 1.0 min and an RT wavelet range from 0.01 to 0.08 min. Isotopes were detected using the isotope peak grouper with an *m/z* tolerance of 5.0 ppm, an RT tolerance of 0.05 min (absolute) and the maximum charge set at 1 and the representative isotope used was the most intense. The resulting peak list was filtered using the peak-filter option (height: 1.0 E^4^ to 1.0 E^7^, data points: 8 to 50, tailing factor: 0.5 to 2.00). Last, using the peak-list rows filter option, features without an MS^2^ spectrum associated were removed. The resulting peak list, containing 283 features, was exported using the GNPS Export/Submit module to an .mgf file and a quantitation table in csv format.

### 4.6. Feature-Based Molecular Networking and Taxonomically Informed Metabolite Annotation

From the .mgf file obtained from the MZmine treatment, an MN was created using the Feature-Based Molecular Networking workflow [13] on the GNPS platform (https://gnps.ucsdhttps://gnps.ucsd.edu.edu). The precursor ion mass and the MS/MS fragment ion tolerances were set to 0.02 and 0.05 Da, respectively. An MN was then created where the edges were filtered above a cosine of 0.6 and more than 3 matches’ peaks. The edges between two nodes were kept in the network if and only if each of the nodes appeared in each other’s respective top 10 most similar nodes. The molecular family size was set to a maximum of 100. The spectra in the network were searched against the GNPS spectral libraries [90]. The work is available at the following link, https://gnps.ucsd.edu/ProteoSAFe/status.jsp?task=9e5e99ef164a4fa0bbe218c73de3ff26. Visualization of the results was carried out in Cytoscape 3.8.0 [91]. The output of the GNPS was used to annotate against the in silico ISDB-DNP [35] and then the script for taxonomically informed metabolite annotation [36] was used to re-rank and clean out the output based on the taxonomy.

## 5. Conclusions

The metabolite profiling workflow based on UHPLC-MS/MS provided a comprehensive survey of the phenolic composition of the methanolic extract of *Stryphnodendron pulcherrimum* leaves. This workflow identified 30 compounds through spectral matching against experimental data from GNPS. Additionally, 19 compounds were putatively assigned via the same spectral matching but against in silico databases. Compounds **1**, **4**, **6**, **13**, **24**, **31**, **42** and **45** have been previously reported in the genus *Stryphnodendron*. Gallic acid (**1**), (epi)gallocatechin (**6**), (epi)gallocatechin gallate (**13**), myricitrin (**31**) and myricetin (**42**) were reported previously in the species by our group [49]. To the best of our knowledge, 44 of these compounds are reported herein for the first time in the species *Stryphnodendron pulcherrimum* and 41 are described for the first time in the genus. The significant and diverse biological activities reported for many of these compounds indicate that this species represents a potentially important biological source of interesting bioactive phenolic compounds.

## Figures and Tables

**Table 1:** UHPLC-MS/MS data of the annotated metabolites in *Stryphnodendron pulcherrimum* leaf extract.

Peak	RT (min)	[M–H]^−^		Molecular Formula	Error (ppm)	MS^2^ Fragments (*m*/*z*)	Compound Name	Spectrum Reference	Group
		Theoretical Mass (*m*/*z*)	Accurate Mass (*m*/*z*)						
1	1.13	169.0137	169.0138	C_7_H_6_O_5_	0.6	125	gallic acid	CCMSLIB00004691622	1
2	1.90	153.0188	153.0191	C_7_H_6_O_4_	2.0	109	protocatechuic acid	CCMSLIB00000578339	1
3	4.61	761.1354	761.1332	C_37_H_30_O_18_	2.9	609, 591, 573, 483, 465, 453, 441, 423, 355, 305, 285, 177, 125	3-*O*-galloyl-(epi)gallocatechin-(epi)gallocatechin	N/A	2
4	5.92	305.0661	305.0677	C_15_H_14_O_7_	5.2	261, 219, 179, 125	gallocatechin	CCMSLIB00000081482	2
5	5.99	479.0826	479.0837	C_21_H_20_O_13_	2.3	435, 389, 313, 250, 169, 125, 121	myricetin 3-sorboside	N/A	3
6	6.07	305.0661	305.0671	C_15_H_14_O_7_	3.3	177, 150, 139, 125, 109	(epi)gallocatechin	CCMSLIB00005787972	2
7	6.54	761.1354	761.1348	C_37_H_30_O_18_	0.8	717, 635, 609, 593, 591, 575, 441, 423, 405, 305, 287, 243, 169, 125	(epi)gallocatechin-3′-*O*-galloyl-(epi)gallocatechin	N/A	2
8	6.95	423.0927	423.0931	C_19_H_20_O_11_	0.9	313, 253, 169, 125	6-*O*-galloylarbutin	N/A	1
9	7.30	483.0775	483.0786	C_20_H_20_O_14_	2.3	331, 313, 287, 271, 211, 169, 125	[3,4,5-trihydroxy-6-(3,4,5-trihydroxybenzoyl)oxyoxan-2-yl]methyl 3,4,5-trihydroxybenzoate Isomer	CCMSLIB00000845139	1
10	7.41	483.0775	483.0792	C_20_H_20_O_14_	3.5	331, 313, 287, 271, 211, 169, 125	[3,4,5-trihydroxy-6-(3,4,5-trihydroxybenzoyl)oxyoxan-2-yl]methyl 3,4,5-trihydroxybenzoate Isomer	CCMSLIB00000845139	1
11	8.28	483.0775	483.0773	C_20_H_20_O_14_	0.4	169, 125	gallic acid *O*-galloyl glucoside	N/A	1
12	9.32	595.1663	595.1663	C_27_H_32_O_15_	0.0	415, 385, 355, 343, 325, 313, 271, 235, 193, 119	3,5,7-trihydroxyflavanone-5-*O*-[galactopyranosyl-glucopyranoside]	N/A	2
13	9.47	457.0771	457.0773	C_22_H_18_O_11_	0.4	305, 261, 219, 179, 169, 125	(epi)gallocatechin gallate	CCMSLIB00004702014	2
14	9.77	467.0826	467.0822	C_20_H_20_O_13_	0.9	449, 423, 315, 313, 193, 169, 152, 125, 109	3,4,5-trihydroxybenzaldehyde-3-*O*-[6-*O*-(3,4,5-trihydroxybenzoyl)-glucopyranoside] Isomer	N/A	1
15	10.18	467.0826	467.0835	C_20_H_20_O_13_	1.9	449, 423, 315, 313, 169, 152, 125, 109	3,4,5-trihydroxybenzaldehyde-3-*O*-[6-*O*-(3,4,5-trihydroxybenzoyl)-glucopyranoside] Isomer	N/A	1
16	10.44	465.1033	465.1039	C_21_H_22_O_12_	1.3	447, 421, 357, 313, 285, 169, 151, 125	taxifolin-4′-glucoside	N/A	2
17	10.51	625.1405	625.1414	C_27_H_30_O_17_	1.4	373, 316, 287, 271, 259, 242, 214, 178, 151, 116	myricetin-3-*O*-hexosyl-deoxyhexoside	CCMSLIB00004678839	3
18	10.54	457.0771	457.0767	C_22_H_18_O_11_	0.9	411, 383, 331, 305, 169, 125	gallocatechin gallate	CCMSLIB00004702014	2
19	11.55	479.0826	479.0824	C_21_H_20_O_13_	0.4	316, 287, 271, 259, 243, 214, 151, 124	myricetin 3-galactoside Isomer	CCMSLIB00004706173	3
20	11.86	479.0826	479.0826	C_21_H_20_O_13_	0.0	316, 287, 271, 259, 243, 214, 151, 124	myricetin 3-galactoside Isomer	CCMSLIB00004706173	3
21	11.91	477.1033	477.1038	C_22_H_22_O_12_	1.0	316, 313, 271, 169, 163, 151, 125, 119	*O*-coumaroyl *O*-galloyl-hexoside	N/A	1
22	12.23	761.1354	761.1372	C_37_H_30_O_18_	2.4	609, 593, 591, 575, 545, 483, 465, 457, 441, 423, 305, 287, 243, 177, 125	3-*O*-galloyl-(epi)gallocatechin-(epi)gallocatechin	N/A	2
23	12.40	595.1299	595.1306	C_26_H_28_O_16_	1.2	431, 355, 316, 271, 219, 179, 151, 100	myricetin-3-*O*-deoxyhexosyl-pentoside	CCMSLIB00004678827	3
24	12.41	471.0927	471.0918	C_23_H_20_O_11_	1.9	429, 347, 305, 287, 183	3*-O*-galloyl-4′-*O*-methylepigallocatechin	N/A	2
25	12.86	495.1503	495.1510	C_23_H_28_O_12_	1.4	313, 181, 179, 169, 151, 125	(3,4,5-trihydroxy-6-(4-(1-hydroxypropan-2-yl)-2-methoxyphenoxy)tetrahydro-2H-pyran-2-yl)methyl 3,4,5-trihydroxybenzoate	N/A	1
26	12.88	493.1346	493.1359	C_23_H_26_O_12_	2.6	313, 181, 179, 169, 151, 125	(3,4,5-trihydroxy-6-(2-hydroxy-4-(3-oxobutyl)phenoxy)tetrahydro-2H-pyran-2-yl)methyl 3,4,5-trihydroxybenzoate	N/A	1
27	12.95	477.1033	477.1041	C_22_H_22_O_12_	1.7	433, 313, 253, 241, 211, 169, 163, 151, 125, 119, 107	methoxy-quercetin-3-*O*-hexoside	CCMSLIB00004678842	3
28	12.97	609.1456	609.1455	C_27_H_30_O_16_	0.2	495, 415, 373, 300, 271, 255, 243, 151, 119	quercetin 3-*O*-neohesperidoside	CCMSLIB00004679290	3
29	13.06	449.0720	449.0724	C_20_H_18_O_12_	0.9	359, 316, 287, 210, 178, 151	myricetin-3-xyloside Isomer	CCMSLIB00000222475	3
30	13.19	449.0720	449.0727	C_20_H_18_O_12_	1.6	316, 287, 271, 214, 178, 151	myricetin-3-xyloside Isomer	CCMSLIB00000222475	3
31	13.35	463.0877	463.0923	C_21_H_20_O_12_	9.9	316, 287, 271, 179, 151, 107	myricitrin Isomer	CCMSLIB00004718497	3
32	13.56	479.1553	479.1566	C_23_H_28_O_11_	2.7	313, 169, 165, 151, 125, 123	3,5-dihydroxy-2-(4-hydroxy-2-isopropyl-5-methylphenoxy)-6-(hydroxymethyl)tetrahydro-2H-pyran-4-yl-3,4,5-trihydroxybenzoate	N/A	1
33	13.64	615.0986	615.0985	C_28_H_24_O_16_	0.2	463, 301, 169	[(2S,3R,4S,5R,6R)-2-[2-(3,4-dihydroxyphenyl)-5,7-dihydroxy-4-oxochromen-3-yl]oxy-4,5-dihydroxy-6-(hydroxymethyl)oxan-3-yl] 3,4,5-trihydroxybenzoate	CCMSLIB00004706250	3
34	13.67	463.0877	463.0869	C_21_H_20_O_12_	1.7	316, 300, 287, 271, 243, 178, 151	myricetin-3-*O*-rhamnopyranoside	CCMSLIB00005463729	3
35	14.05	463.0877	463.0869	C_21_H_20_O_12_	1.7	316, 300, 259, 218, 179, 117	myricitrin Isomer	CCMSLIB00004706620	3
36	14.14	463.0877	463.0913	C_21_H_20_O_12_	7.8	300, 271, 255, 179, 151, 104	isoquercitrin	CCMSLIB00000077231	3
37	14.68	433.0771	433.0759	C_20_H_18_O_11_	2.8	387, 348, 300, 249, 217, 178, 113	avicularin	CCMSLIB00004706151	3
38	14.71	479.1553	479.1549	C_23_H_28_O_11_	0.8	313, 300, 169, 151, 123	6″-*O*-galloylepirhododendrin	N/A	1
39	14.87	477.1033	477.1037	C_22_H_22_O_12_	0.8	405, 316, 300	3′,5,7,8-tetrahydroxy-4′-methoxyflavone-8-*O-*glucopyranoside	N/A	3
40	15.19	463.0877	463.0906	C_21_H_20_O_12_	6.2	391, 301, 272, 239, 151	quercetin-4-glucoside	CCMSLIB00004683609	3
41	16.05	447.0927	447.0951	C_21_H_20_O_11_	5.3	300, 271, 255, 243, 151	quercitrin	CCMSLIB00004679288	3
42	16.22	317.0297	317.0315	C_15_H_10_O_8_	5.7	178, 151, 137, 107	myricetin	CCMSLIB00004705572	3
43	16.53	479.1553	479.1557	C_23_H_28_O_11_	0.8	169, 165, 151, 125	querglanin	N/A	1
44	17.60	433.1135	433.1137	C_21_H_22_O_10_	0.5	313, 169, 151, 125, 119	phlorizin chalcone	N/A	1
45	17.88	447.0927	447.0936	C_21_H_20_O_11_	2.0	432, 405, 285, 199, 1 75, 151, 113	luteolin-4′-*O*-glucoside	CCMSLIB00004720065	3
46	18.25	431.0978	431.0956	C_21_H_20_O_10_	5.1	314, 285, 255, 227, 124	kaempferol-7-*O*-deoxyhexoside	CCMSLIB00004678854	3
47	20.30	301.0348	301.0354	C_15_H_10_O_7_	2.0	257, 178, 145, 116	tricetin	CCMSLIB00004691766	3
48	20.41	285.0399	285.0404	C_15_H_10_O_6_	1.7	251, 183, 116	3′,4′,5,7-tetrahydroxyflavone	CCMSLIB00004691238	3
49	21.97	315.0505	315.0506	C_16_H_12_O_7_	0.3	300, 231, 188, 116	3′-*O-*methylquercetin	CCMSLIB00000081595	3

Note: 1: gallic acid group; 2: flavan-3-ols group; 3: flavones group. N/A: not available, meaning this annotation is a spectral match against the in silico database.

## Data Availability

All support data used in this study are available from the authors.

## Supplementary Materials

The following are available online at https://www.mdpi.com/article/10.3390/metabo11050281/s1, Compounds annotated in GNPS; Table S1. In-house database including 74 compounds reported in the literature from *Stryphnodendron*. Figure S1. Matches between spectrum MS/MS from G1. Figure S2. Matches between spectrum MS/MS from G2. Figure S3. Matches between spectrum MS/MS from G3. Figure S4. Fragmentation scheme of (epi)gallocatechin gallate (**13**). Figure S5. Fragmentation scheme of O-coumaroyl O-galloyl-hexoside (**21**). Figure S6. Fragmentation scheme of (3,4,5-trihydroxy-6-(4-(1-hydroxypropan-2-yl)-2-methoxyphenoxy)tetrahydro-2H-pyran-2-yl)methyl 3,4,5-trihydroxybenzoate (**25**). Figure S7. Fragmentation scheme of myricitrin (**31**). Figure S8. Fragmentation scheme of quercitrin (**41**).
