# Supplementary material for: Feature-Based Molecular Network-Guided Dereplication of Natural Bioactive Products from Leaves of Stryphnodendron pulcherrimum (Willd.) Hochr
Source: Metabolites. 2021 Apr 29;11(5):281. doi: 10.3390/metabo11050281 (PMC8147077; doi:10.3390/metabo11050281)
Supplement: Supplementary file 1 [file metabolites-11-00281-s001.zip › metabolites-1129666-SI.pdf]

Supplementary Material

for

Feature-Based Molecular Network-guided dereplication of natural bioactive products from leaves of *Stryphnodendron pulcherrimum* (Willd.) Hochr

Paulo Gomes [1ǂ], Luis Quirós-Guerrero [2,3,ǂ], Consuelo Silva [1,4], Sônia Pamplona [1], Jean A. Boutin [5,6], Marcos Eberlin [7], Jean-Luc Wolfender [2,3] and Milton Silva [1,*]

[1] Laboratory of Liquid Chromatography, Institute of Exact and Natural Sciences, Federal University of Pará, Belem, Brazil; wenderufpa@hotmail.com (P.G.); yumikoyoshioka@yahoo.com.br (Y.S.); sgpamplona@yahoo.com.br (S.P.);

[2] School of Pharmaceutical Sciences, University de Geneva, CMU, Rue Michel-Servet 1, CH-1206 Geneva 4, Switzerland; Luis.Guerrero@unige.ch (L.Q.-G.); Jean-Luc.Wolfender (J.-L.W.);

[3] Institute of Pharmaceutical Sciences of Western Switzerland, University of Geneva, CMU, Rue Michel Servet 1, 1211 Geneva 4, Switzerland.

[4] Pharmaceutical Science Post-Graduation Program, Faculty of Pharmacy, Federal University of Pará, 66075-110, Pará, Brazil.

[5] Institut de Recherches Internationales Servier, 50 rue Carnot, 92284 Suresnes, France.

[6] Present address: PHARMADEV (Pharmacochimie et biologie pour le développement), Université Toulouse 3 Paul Sabatier, Faculté de Pharmacie, 35 chemin des Maraîchers - 31062 Toulouse Cedex 9, France; ja.boutin.pro@gmail.com (J.A.B.)

[7] MackMass Laboratory, Mackenzie Presbyterian University, School of Engineering – PPGEMN, São Paulo, SP, 01302-907, Brazil; marcos.eberlin@mackenzie.br (M.B.)

* Correspondence: yumilto@yahoo.com.br; Tel.: +55-913201736; ǂequal contribution

List of abbreviations

UHPLC-MS/MS: ultra-high performance liquid chromatography-mass spectrometry.
GNPS: Global Natural Products Social
NMR: Nuclear magnetic resonance
FBMN: Feature-Based Molecular Network
SPE: Solid Phase Extract
MS: Mass Spectrometry
MN: Molecular network
MS^1: Level one spectra, ionized molecule
MS^2: Tandem mass spectrometry spectra, often designated as MS/MS (fragmentation spectra)
DNP: Dictionary of Natural Products
MF: Molecular Formula
RT: Retention time
QM: Quinone Methide
HRF: Heterolytic Ring Fission
RDA: Retro Diels-Alder
ISDB: *In-Silico* Database
HCOE: Hydro cetonic extract
ESI: Electrospray ionization
BEH: Ethylene Bridged Hybrid
DDA: Data Dependent Acquisition
NCE: Normalized Collision Energy
ADAP: Automated Data Analysis Pipeline

Table S1. *In house* database for the genus *Stryphnodendron.*

ID	Compound	MF	[M-H]⁻Calculated	InChl	Species	Reference
1	4-Hydroxybenzaldehyde	$C_7H_6O_2$	121.0290	InChI=1S/C7H6O2/c8-5-6-1-3-7(9)4-2-6/h1-5,9H	SP*	[22]
2	Salicylic acid	$C_7H_6O_3$	137.0239	InChI=1S/C7H6O3/c8-6-4-2-1-3-5(6)7(9)10/h1-4,8H,(H,9,10)	SP*	[22]
3	Gentile acid	$C_7H_6O_4$	153.0188	InChI=1S/C7H6O4/c8-4-1-2-6(9)5(3-4)7(10)11/h1-3,8-9H,(H,10,11)	SP*	[22]
4	Gallic acid	$C_7H_6O_5$	169.0137	InChI=1S/C7H6O5/c8-4-1-3(7(11)12)2-5(9)6(4)10/h1-2,8-10H,(H,11,12)	SA, SP, SR, SP*	[2–7,11,22–24]
5	Caffeic acid	$C_9H_8O_4$	179.0344	InChI=1S/C9H8O4/c10-7-3-1-6(5-8(7)11)2-4-9(12)13/h1-5,10-11H,(H,12,13)	SR	[60]
6	Luteolin	$C_{15}H_{10}O_6$	285.0399	InChI=1S/C15H10O6/c16-8-4-11(19)15-12(20)6-13(21-14(15)5-8)7-1-2-9(17)10(18)3-7/h1-6,16-19H	SP*	[22]
7	Catechin	$C_{15}H_{14}O_6$	289.0712	InChI=1S/C15H14O6/c16-8-4-11(18)9-6-13(20)15(21-14(9)5-8)7-1-2-10(17)12(19)3-7/h1-5,13,15-20H,6H2	SA, SR, SP*	[3,6,8,22,23]
8	Epicatechin	$C_{15}H_{14}O_6$	289.0712	InChI=1S/C15H14O6/c16-8-4-11(18)9-6-13(20)15(21-14(9)5-8)7-1-2-10(17)12(19)3-7/h1-5,13,15-20H,6H2	SA	[8]
9	Robinetinidol	$C_{15}H_{14}O_6$	289.0712	InChI=1S/C15H14O6/c16-9-2-1-7-3-12(19)15(21-13(7)6-9)8-4-10(17)14(20)11(18)5-8/h1-2,4-6,12,15-20H,3H2/t12-,15+/m0/s1	SA	[7,24]
10	Quercetin	$C_{15}H_{10}O_7$	301.0348	InChI=1S/C15H10O7/c16-7-4-10(19)12-11(5-7)22-15(14(21)13(12)20)6-1-2-8(17)9(18)3-6/h1-5,16-19,21H	SP*	[22]
11	Gallocatechin	$C_{15}H_{14}O_7$	305.0661	InChI=1S/C15H14O7/c16-7-3-9(17)8-5-12(20)15(22-13(8)4-7)6-1-10(18)14(21)11(19)2-6/h1-4,12,15-21H,5H2/t12-,15+/m0/s1	SA, SP, SR	[2–6,8,9,11]

12	Epigallocatechin	$C_{15}H_{14}O_7$	305.0661	InChI=1S/C15H14O7/c16-7-3-9(17)8-5-12(20)15(22-13(8)4-7)6-1-10(18)14(21)11(19)2-6/h1-4,12,15-21H,5H2/t12-,15-/m1/s1	SA, SP, SR	[2–9,11]
13	Myricetin	$C_{15}H_{10}O_8$	317.0297	InChI=1S/C15H10O8/c16-6-3-7(17)11-10(4-6)23-15(14(22)13(11)21)5-1-8(18)12(20)9(19)2-5/h1-4,16-20,22H	SP*	[22]
14	4′-*O*-methylgallocatechin	$C_{16}H_{16}O_7$	319.0818	InChI=1S/C16H16O7/c1-22-16-11(19)2-7(3-12(16)20)15-13(21)6-9-10(18)4-8(17)5-14(9)23-15/h2-5,13,15,17-21H,6H2,1H3/t13-,15+/m0/s1	SA, SP	[2–6,8,9,11]
15	4′-*O*-methylepigallocatechin	$C_{16}H_{16}O_7$	319.0818	InChI=1S/C23H20O11/c1-32-22-16(28)2-9(3-17(22)29)21-19(8-12-13(25)6-11(24)7-18(12)33-21)34-23(31)10-4-14(26)20(30)15(27)5-10/h2-7,19,21,24-30H,8H2,1H3/t19-,21-/m1/s1	SA	[2–9,11]
16	di-O-hexoside	$C_{12}H_{22}O_{11}$	341.1091	InChI=1S/C12H22O11/c13-1-3-5(15)7(17)9(19)11(21-3)23-12-10(20)8(18)6(16)4(2-14)22-12/h3-20H,1-2H2	SA	[11]
17	NI	$C_{22}H_{18}O_9$	425.0872	N/A	SA	[11]
18	Luteolin-4′-*O*-hexoside	$C_{21}H_{20}O_{11}$	447.0927	InChI=1S/C21H20O11/c22-7-16-18(27)19(28)20(29)21(32-16)31-13-2-1-8(3-10(13)24)14-6-12(26)17-11(25)4-9(23)5-15(17)30-14/h1-6,16,18-25,27-29H,7H2/t16-,18-,19+,20-,21-/m1/s1	SA	[25]
19	Quercetin-3-O-deoxyhexoside	$C_{21}H_{20}O_{11}$	447.0927	InChI=1S/C27H30O15/c1-8-17(32)20(35)22(37)26(38-8)42-25-21(36)18(33)9(2)39-27(25)41-24-19(34)16-14(31)6-11(28)7-15(16)40-23(24)10-3-4-12(29)13(30)5-10/h3-9,17-18,20-22,25-33,35-37H,1-2H3	SP*	[22]
20	Epigallocatechin 3-*O*-(3-methoxy-4-hydroxy)benzoate	$C_{23}H_{20}O_{10}$	455.0978	InChI=1S/C23H20O10/c1-31-19-6-10(2-3-14(19)25)23(30)33-20-9-13-15(26)7-12(24)8-18(13)32-22(20)11-4-16(27)21(29)17(28)5-11/h2-8,20,22,24-29H,9H2,1H3/t20?,22-/m1/s1	SA, SP*	[2,9,22]

21	Epigallocatechin 3-*O*-gallate	$C_{22}H_{18}O_{11}$	457.0771	InChI=1S/C22H18O11/c23-10-5-12(24)11-7-18(33-22(31)9-3-15(27)20(30)16(28)4-9)21(32-17(11)6-10)8-1-13(25)19(29)14(26)2-8/h1-6,18,21,23-30H,7H2/t18-,21-/m1/s1	SA, SR, SP*	[3,5–7,9,11,22,26]
22	Myricitrin	$C_{21}H_{20}O_{12}$	463.0877	InChI=1S/C21H20O12/c1-6-14(26)17(29)18(30)21(31-6)33-20-16(28)13-9(23)4-8(22)5-12(13)32-19(20)7-2-10(24)15(27)11(25)3-7/h2-6,14,17-18,21-27,29-30H,1H3/t6-,14-,17+,18+,21-/m0/s1	SP*	[22]
23	Epigallocatechin 3-*O*-methylgallate	$C_{23}H_{20}O_{11}$	471.0927	InChI=1S/C23H20O11/c1-32-18-5-10(4-16(28)21(18)30)23(31)34-19-8-12-13(25)6-11(24)7-17(12)33-22(19)9-2-14(26)20(29)15(27)3-9/h2-7,19,22,24-30H,8H2,1H3/t19-,22-/m1/s1	SA	[7]
24	4′-*O*-methylepigallocatechin-3-*O*-gallate	$C_{23}H_{20}O_{11}$	471.0927	InChI=1S/C23H20O11/c1-32-22-16(28)2-9(3-17(22)29)21-19(8-12-13(25)6-11(24)7-18(12)33-21)34-23(31)10-4-14(26)20(30)15(27)5-10/h2-7,19,21,24-30H,8H2,1H3/t19-,21-/m1/s1	SA	[5]
25	Myricetin-3-O-galactoside	$C_{21}H_{20}O_{13}$	479.0826	InChI=1S/C21H20O13/c22-5-12-15(28)17(30)18(31)21(33-12)34-20-16(29)13-8(24)3-7(23)4-11(13)32-19(20)6-1-9(25)14(27)10(26)2-6/h1-4,12,15,17-18,21-28,30-31H,5H2/t12-,15+,17+,18-,21+/m1/s1	SP*	[22]
26	C-hexosyl O-pentosyl 5,7-dihydroxychromone	$C_{21}H_{26}O_{13}$	485.1299	N/A	SA	[11]
27	Fisetinidol-(4*α*→8)-gallocatechin	$C_{26}H_{30}O_{12}$	533.1659	InChI=1S/C30H26O12/c31-13-2-3-14-23(8-13)41-29(11-1-4-16(32)18(34)5-11)27(40)24(14)25-19(35)10-17(33)15-9-22(38)28(42-30(15)25)12-6-20(36)26(39)21(37)7-12/h1-8,10,22,24,27-29,31-40H,9H2/t22-,24+,27-,28-,29+/m0/s1	SP	[2]
28	Fisetinidol-(4β→8)-gallocatechin	$C_{26}H_{30}O_{12}$	533.1659	InChI=1S/C30H26O12/c31-13-2-3-14-23(8-13)41-29(11-1-4-16(32)18(34)5-11)27(40)24(14)25-	SP	[2]

				19(35)10-17(33)15-9-22(38)28(42-30(15)25)12-6-20(36)26(39)21(37)7-12/h1-8,10,22,24,27-29,31-40H,9H2/t22-,24-,27-,28-,29+/m0/s1		
29	NI	$C_{26}H_{32}O_{12}$	535.1827	N/A	SA	[11]
30	Procyanidin dimer (B type)	$C_{30}H_{26}O_{12}$	577.1368	InChI=1S/C30H26O12/c31-13-7-20(37)24-23(8-13)41-29(12-2-4-16(33)19(36)6-12)27(40)26(24)25-21(38)10-17(34)14-9-22(39)28(42-30(14)25)11-1-3-15(32)18(35)5-11/h1-8,10,22,26-29,31-40H,9H2	SA	[11]
31	Luteolin-7-*O*-hexoxyl(6″→2′″)pentoside	$C_{26}H_{28}O_{15}$	579.1350	InChI=1S/C26H28O15/c27-11-2-1-9(3-12(11)28)16-6-14(30)19-13(29)4-10(5-17(19)40-16)39-26-24(36)22(34)21(33)18(41-26)8-38-25-23(35)20(32)15(31)7-37-25/h1-6,15,18,20-29,31-36H,7-8H2/t15?,18-,20?,21-,22+,23?,24-,25?,26-/m1/s1	SA	[25]
32	Robinetinidol-(4*α*→8)-epigallocatechin	$C_{30}H_{26}O_{13}$	593.1295	InChI=1S/C30H26O13/c31-12-1-2-13-22(7-12)42-29(11-5-19(36)26(40)20(37)6-11)27(41)23(13)24-16(33)9-15(32)14-8-21(38)28(43-30(14)24)10-3-17(34)25(39)18(35)4-10/h1-7,9,21,23,27-29,31-41H,8H2/t21-,23-,27+,28-,29-/m1/s1	SA	[7,24]
33	Robinetinidol-(4β→8)-epigallocatechin	$C_{30}H_{26}O_{13}$	593.1295	InChI=1S/C30H26O13/c31-12-1-2-13-22(7-12)42-29(11-5-19(36)26(40)20(37)6-11)27(41)23(13)24-16(33)9-15(32)14-8-21(38)28(43-30(14)24)10-3-17(34)25(39)18(35)4-10/h1-7,9,21,23,27-29,31-41H,8H2/t21-,23+,27+,28-,29-/m1/s1	SA	[24]
34	Robinetinidol-(4*α*→6)-gallocatechin	$C_{30}H_{26}O_{13}$	593.1295	InChI=1S/C30H26O13/c31-12-1-2-13-21(7-12)43-30(11-5-18(35)27(40)19(36)6-11)28(41)23(13)24-15(32)9-22-14(25(24)38)8-20(37)29(42-22)10-3-16(33)26(39)17(34)4-10/h1-7,9,20,23,28-41H,8H2/t20-,23?,28-,29-,30+/m0/s1	SA	[24]
35	Robinetinidol-(4*α*→6)-epigallocatechin	$C_{30}H_{26}O_{13}$	593.1295	InChI=1S/C30H26O13/c31-12-1-2-13-21(7-12)43-30(11-5-18(35)27(40)19(36)6-11)28(41)23(13)24-	SA	[24]

				15(32)9-22-14(25(24)38)8-20(37)29(42-22)10-3-16(33)26(39)17(34)4-10/h1-7,9,20,23,28-41H,8H2/t20-,23?,28+,29-,30-/m1/s1		
36	Robinetinidol-(4α→8)-gallocatechin	$C_{30}H_{26}O_{13}$	593.1295	InChI=1S/C30H26O13/c31-12-1-2-13-22(7-12)42-29(11-5-19(36)26(40)20(37)6-11)27(41)23(13)24-16(33)9-15(32)14-8-21(38)28(43-30(14)24)10-3-17(34)25(39)18(35)4-10/h1-7,9,21,23,27-29,31-41H,8H2/t21-,23?,27-,28-,29+/m0/s1	SA	[24]
37	Catechin-gallocatechin	$C_{30}H_{26}O_{13}$	593.1295	InChI=1S/C30H26O13/c31-12-6-17(35)23-22(7-12)42-29(10-1-2-14(32)16(34)3-10)27(41)25(23)24-18(36)9-15(33)13-8-21(39)28(43-30(13)24)11-4-19(37)26(40)20(38)5-11/h1-7,9,21,25,27-29,31-41H,8H2/t21-,25?,27?,28-,29?/m0/s1	SP*	[22]
38	Prorobinetidin-prodelphinidin dimer (B type)	$C_{30}H_{26}O_{13}$	593.1299	N/A	SA	[11]
39	Robinetinidol-[4β→6(8)]-gallocatechin	$C_{30}H_{26}O_{13}$	593.1312	InChI=1S/C30H26O13/c31-12-1-2-13-21(7-12)43-30(11-5-18(35)27(40)19(36)6-11)28(41)23(13)24-15(32)9-22-14(25(24)38)8-20(37)29(42-22)10-3-16(33)26(39)17(34)4-10/h1-7,9,20,23,28-41H,8H2/t20?,23?,28-,29?,30+/m0/s1	SA	[24]
40	Procyanidin-prodelphinidin dimer (B type)	$C_{30}H_{26}O_{13}$	593.1312	N/A	SA	[11]
41	Procyanidin-prodelphinidin dimer (B type)	$C_{30}H_{26}O_{13}$	593.1312	N/A	SA	[11]
42	Capreoside	$C_{27}H_{30}O_{15}$	595.1506	InChI=1S/C27H30O15/c1-37-15-3-2-10(4-12(15)29)16-7-14(31)20-13(30)5-11(6-17(20)40-16)39-27-25(36)23(34)22(33)19(42-27)9-38-26-24(35)21(32)18(8-28)41-26/h2-7,18-19,21-30,32-36H,8-9H2,1H3/t18-,19+,21-,22+,23-,24+,25+,26+,27+/m0/s1	SA	[25]

43	Robinetinidol-4′-*O*-methylepigallocatechin	$C_{31}H_{28}O_{13}$	607.1452	InChI=1S/C31H28O13/c1-42-31-20(37)6-12(7-21(31)38)29-27(41)24(14-3-2-13(32)8-23(14)43-29)25-17(34)10-16(33)15-9-22(39)28(44-30(15)25)11-4-18(35)26(40)19(36)5-11/h2-8,10,22,24,27-29,32-41H,9H2,1H3/t22-,24?,27+,28-,29-/m1/s1	SA	[7,24]
44	Prodelphinidin dimer (B-type)	$C_{30}H_{26}O_{14}$	609.1227	N/A	SA, SP*	[11,22]
45	Epigallocatechin-(4β→8)-gallocatechin	$C_{30}H_{26}O_{14}$	609.1244	InChI=1S/C30H26O14/c31-11-5-14(33)22-21(6-11)43-29(10-3-18(37)26(41)19(38)4-10)27(42)24(22)23-15(34)8-13(32)12-7-20(39)28(44-30(12)23)9-1-16(35)25(40)17(36)2-9/h1-6,8,20,24,27-29,31-42H,7H2/t20-,24+,27+,28-,29+/m0/s1	SA, SP	[2,9]
46	Epigallocatechin-(4β→8)-epigallocatechin	$C_{30}H_{26}O_{14}$	609.1244	InChI=1S/C30H26O14/c31-11-5-14(33)22-21(6-11)43-29(10-3-18(37)26(41)19(38)4-10)27(42)24(22)23-15(34)8-13(32)12-7-20(39)28(44-30(12)23)9-1-16(35)25(40)17(36)2-9/h1-6,8,20,24,27-29,31-42H,7H2/t20-,24-,27-,28-,29-/m1/s1	SA	[5,7,9]
47	Epigallocatechin-(4β→6)-epigallocatechin	$C_{30}H_{26}O_{14}$	609.1244	InChI=1S/C30H26O14/c31-11-5-13(32)22-21(6-11)44-30(10-3-17(36)27(41)18(37)4-10)28(42)24(22)23-14(33)8-20-12(25(23)39)7-19(38)29(43-20)9-1-15(34)26(40)16(35)2-9/h1-6,8,19,24,28-42H,7H2/t19-,24-,28-,29-,30-/m1/s1	SA	[5,7,9]
48	Prodelphinidin dimer (B-type)	$C_{30}H_{26}O_{14}$	609.1244	N/A	SA	[11]
49	Prodelphinidin dimer (B-type)	$C_{30}H_{26}O_{14}$	609.1244	N/A	SA	[11]
50	Prodelphinidin dimer (B-type)	$C_{30}H_{26}O_{14}$	609.1244	N/A	SA	[11]
51	Prodelphinidin dimer (B-type)	$C_{30}H_{26}O_{14}$	609.1244	N/A	SA	[11]
52	Rutin	$C_{27}H_{30}O_{16}$	609.1456	InChI=1S/C27H30O16/c1-8-17(32)20(35)22(37)26(40-8)39-7-15-18(33)21(36)23(38)27(42-15)43-25-19(34)16-	SR	[23]

				13(31)5-10(28)6-14(16)41-24(25)9-2-3-11(29)12(30)4-9/h2-6,8,15,17-18,20-23,26-33,35-38H,7H2,1H3/t8-,15+,17-,18+,20+,21-,22+,23+,26+,27-/m1/s1		
53	Epigallocatechin 3-*O*-(3,5-dimethyl)gallate	$C_{24}H_{22}O_{19}$	613.0977		SA	[2,9]
54	4′-*O*-methylrobinetinidol-(4*α*→8)-4′-*O*-methylgallocatechin	$C_{32}H_{30}O_{13}$	621.1608	InChI=1S/C32H30O13/c1-42-31-19(36)5-12(6-20(31)37)28-23(40)10-16-17(34)11-18(35)26(30(16)45-28)25-15-4-3-14(33)9-24(15)44-29(27(25)41)13-7-21(38)32(43-2)22(39)8-13/h3-9,11,23,25,27-29,33-41H,10H2,1-2H3/t23-,25-,27-,28-,29+/m0/s1	SA	[2,9]
55	4′-*O*-methylrobinetinidol-(4*α*→8)-4′-*O*-methylepigallocatechin	$C_{32}H_{30}O_{13}$	621.1608	InChI=1S/C32H30O13/c1-42-31-19(36)5-12(6-20(31)37)28-23(40)10-16-17(34)11-18(35)26(30(16)45-28)25-15-4-3-14(33)9-24(15)44-29(27(25)41)13-7-21(38)32(43-2)22(39)8-13/h3-9,11,23,25,27-29,33-41H,10H2,1-2H3/t23-,25+,27+,28-,29-/m1/s1	SA	[2,9]
56	4′-*O*-methylrobinetinidol-(4β→6)-4′-*O*-methylgallocatechin	$C_{32}H_{30}O_{13}$	621.1608	InChI=1S/C32H30O13/c1-42-31-18(35)5-12(6-19(31)36)29-22(39)10-16-24(44-29)11-17(34)26(27(16)40)25-15-4-3-14(33)9-23(15)45-30(28(25)41)13-7-20(37)32(43-2)21(38)8-13/h3-9,11,22,25,28-30,33-41H,10H2,1-2H3/t22-,25+,28-,29-,30+/m0/s1	SA, SP	[2,27]
57	4′-*O*-methylepigallocatechin-epigallocatechin	$C_{31}H_{28}O_{14}$	623.1401	InChI=1S/C31H28O14/c1-43-31-19(38)4-11(5-20(31)39)29-27(42)25(23-15(34)6-12(32)7-22(23)44-29)24-16(35)9-14(33)13-8-21(40)28(45-30(13)24)10-2-17(36)26(41)18(37)3-10/h2-7,9,21,25,27-29,32-42H,8H2,1H3/t21-,25?,27-,28-,29-/m1/s1	SA	[5]

58	4′-*O*-methylepigallocatechin-4′-*O*-methylepigallocatechin	$C_{32}H_{30}O_{14}$	637.1557	InChI=1S/C32H30O14/c1-43-31-18(37)3-11(4-19(31)38)28-22(41)9-14-15(34)10-17(36)25(30(14)46-28)26-24-16(35)7-13(33)8-23(24)45-29(27(26)42)12-5-20(39)32(44-2)21(40)6-12/h3-8,10,22,26-29,33-42H,9H2,1-2H3/t22-,26?,27-,28-,29-/m1/s1	SA	[7]
59	NI	$C_{33}H_{38}O_{17}$	705.2042	N/A	SA	[11]
60	Gallocatechin-(4*α*→8)-epigallocatechin 3-*O*-(4-hydroxy)benzoate	$C_{37}H_{30}O_{16}$	729.1456	InChI=1S/C37H30O16/c38-16-3-1-13(2-4-16)37(50)52-27-11-18-19(40)12-21(42)29(36(18)53-34(27)14-5-22(43)31(47)23(44)6-14)30-28-20(41)9-17(39)10-26(28)51-35(33(30)49)15-7-24(45)32(48)25(46)8-15/h1-10,12,27,30,33-35,38-49H,11H2/t27-,30+,33+,34-,35+/m1/s1	SA	[7,9]
61	Epigallocatechin-(4β→8)-epigallocatechin 3-*O*-(4-hydroxy)benzoate	$C_{37}H_{30}O_{16}$	729.1456	InChI=1S/C37H30O16/c38-16-3-1-13(2-4-16)37(50)52-27-11-18-19(40)12-21(42)29(36(18)53-34(27)14-5-22(43)31(47)23(44)6-14)30-28-20(41)9-17(39)10-26(28)51-35(33(30)49)15-7-24(45)32(48)25(46)8-15/h1-10,12,27,30,33-35,38-49H,11H2/t27-,30-,33-,34-,35-/m1/s1	SA	[7,9]
62	O-benzoyl prodelphinidin dimer (B-type)	$C_{37}H_{30}O_{16}$	729.1456	N/A	SA	[11]
63	Robinetinidol-(4β→8)-epigallocatechin-3-*O*-gallate	$C_{37}H_{30}O_{17}$	745.1405	InChI=1S/C37H30O17/c38-15-1-2-16-26(9-15)52-35(13-5-22(43)31(48)23(44)6-13)33(50)28(16)29-19(40)11-18(39)17-10-27(53-37(51)14-7-24(45)32(49)25(46)8-14)34(54-36(17)29)12-3-20(41)30(47)21(42)4-12/h1-9,11,27-28,33-35,38-50H,10H2/t27-,28-,33+,34-,35-/m1/s1	SA	[24]
64	Robinetinidol-(4*α*→8)-epigallocatechin-3-*O*-gallate	$C_{37}H_{30}O_{17}$	745.1405	InChI=1S/C37H30O17/c38-15-1-2-16-26(9-15)52-35(13-5-22(43)31(48)23(44)6-13)33(50)28(16)29-19(40)11-18(39)17-10-27(53-37(51)14-7-24(45)32(49)25(46)8-14)34(54-36(17)29)12-3-	SA	[24]

				20(41)30(47)21(42)4-12/h1-9,11,27-28,33-35,38-50H,10H2/t27-,28+,33+,34-,35-/m1/s1		
65	O-galloyl prorobinetidin-prodelphinidin dimer (B type)	$C_{37}H_{30}O_{17}$	745.1405	N/A	SA	[11]
66	NI	$C_{37}H_{30}O_{18}$	761.1354	N/A	SA	[11]
67	Gallocatechin-(4β→8)-epigallocatechin 3-*O*-gallate	$C_{37}H_{30}O_{18}$	761.1354	InChI=1S/C37H30O18/c38-14-7-17(40)27-25(8-14)53-35(12-3-21(44)31(49)22(45)4-12)33(51)29(27)28-18(41)10-16(39)15-9-26(54-37(52)13-5-23(46)32(50)24(47)6-13)34(55-36(15)28)11-1-19(42)30(48)20(43)2-11/h1-8,10,26,29,33-35,38-51H,9H2/t26-,29+,33-,34-,35-/m0/s1	SA	[9]
68	Epigallocatechin-(4β→8)-epigallocatechin-3-*O*-gallate	$C_{37}H_{30}O_{18}$	761.1354	InChI=1S/C37H30O18/c38-14-7-17(40)27-25(8-14)53-35(12-3-21(44)31(49)22(45)4-12)33(51)29(27)28-18(41)10-16(39)15-9-26(54-37(52)13-5-23(46)32(50)24(47)6-13)34(55-36(15)28)11-1-19(42)30(48)20(43)2-11/h1-8,10,26,29,33-35,38-51H,9H2/t26-,29+,33+,34-,35+/m0/s1	SA	[9]
69	Epigallocatechin-epigallocatechin 3-*O*-gallate	$C_{37}H_{30}O_{18}$	761.1354	InChI=1S/C37H30O18/c38-14-7-17(40)27-25(8-14)53-35(12-3-21(44)31(49)22(45)4-12)33(51)29(27)28-18(41)10-16(39)15-9-26(54-37(52)13-5-23(46)32(50)24(47)6-13)34(55-36(15)28)11-1-19(42)30(48)20(43)2-11/h1-8,10,26,29,33-35,38-51H,9H2/t26-,29+,33+,34-,35+/m0/s1	SA	[5,7,9]
70	O-galloyl prodelphinidin dimer (B-type)	$C_{37}H_{30}O_{18}$	761.1354	N/A	SA	[11]
71	Prodelphinidin trimer (B-type)	$C_{48}H_{38}O_{21}$	903.1873	N/A	SA	[11]

72	Epigallocatechin 3-*O*-gallate-(4β→8)-epigallocatechin 3-*O*-gallate	$C_{44}H_{34}O_{22}$	913.1463	InChI=1S/C44H34O22/c45-17-9-20(47)32-30(10-17)63-40(14-3-24(51)36(58)25(52)4-14)42(66-44(62)16-7-28(55)38(60)29(56)8-16)34(32)33-21(48)12-19(46)18-11-31(64-43(61)15-5-26(53)37(59)27(54)6-15)39(65-41(18)33)13-1-22(49)35(57)23(50)2-13/h1-10,12,31,34,39-40,42,45-60H,11H2/t31-,34+,39-,40+,42+/m0/s1	SA	[5,7,9]
73	4′-*O*-methylepigallocatechin 3-*O*-gallate-epigallocatechin 3-*O*-gallate	$C_{45}H_{36}O_{22}$	927.1620	InChI=1S/C45H36O22/c1-63-42-29(56)4-15(5-30(42)57)40-43(67-45(62)17-8-27(54)38(60)28(55)9-17)35(33-21(48)10-18(46)11-31(33)64-40)34-22(49)13-20(47)19-12-32(65-44(61)16-6-25(52)37(59)26(53)7-16)39(66-41(19)34)14-2-23(50)36(58)24(51)3-14/h2-11,13,32,35,39-40,43,46-60H,12H2,1H3/t32-,35+,39-,40+,43+/m0/s1	SA	[5,7,9]
74	Polymer of with 6 monomers of flavan-3-ols	$C_{105}H_{84}O_{49}$	2127.4003	N/A	SA	[28]

Note: (Data were reported in the literature 1989-2021 – Source: Scopus, Web of Science, Science Direct, Scielo, PubMed, and Google Scholar, as well as the patent offices in Brazil (INPI), United States (USPTO), Europe (EPO) and World Intellectual Property Organization (WIPO). N/A: Not available; SA: *Stryphnodendron adstringens*; SP: *Stryphnodendron polyphyllum*; SR: *Stryphnodendron rotundifolium;* SP*: *Stryphnodendron pulcherrimum.*

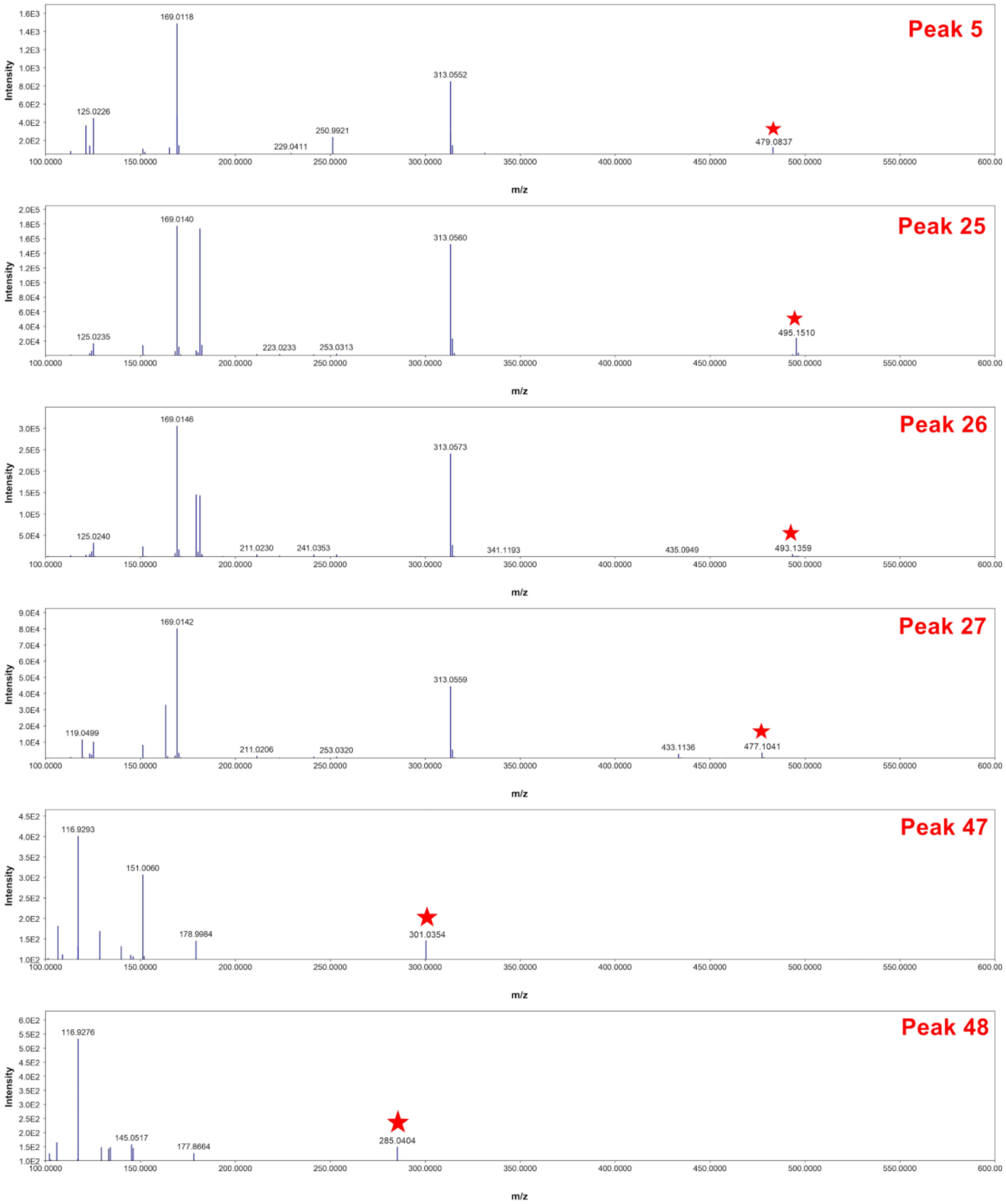

Figure. S1. Some compounds share same fragment-ion on G1 and G3: matches of MS/MS spectra for compounds **25**, **26** gallic acid derivatives (G1) and compounds **5**, **27, 47, 48** flavones derivatives (G3); Red star: parental ion

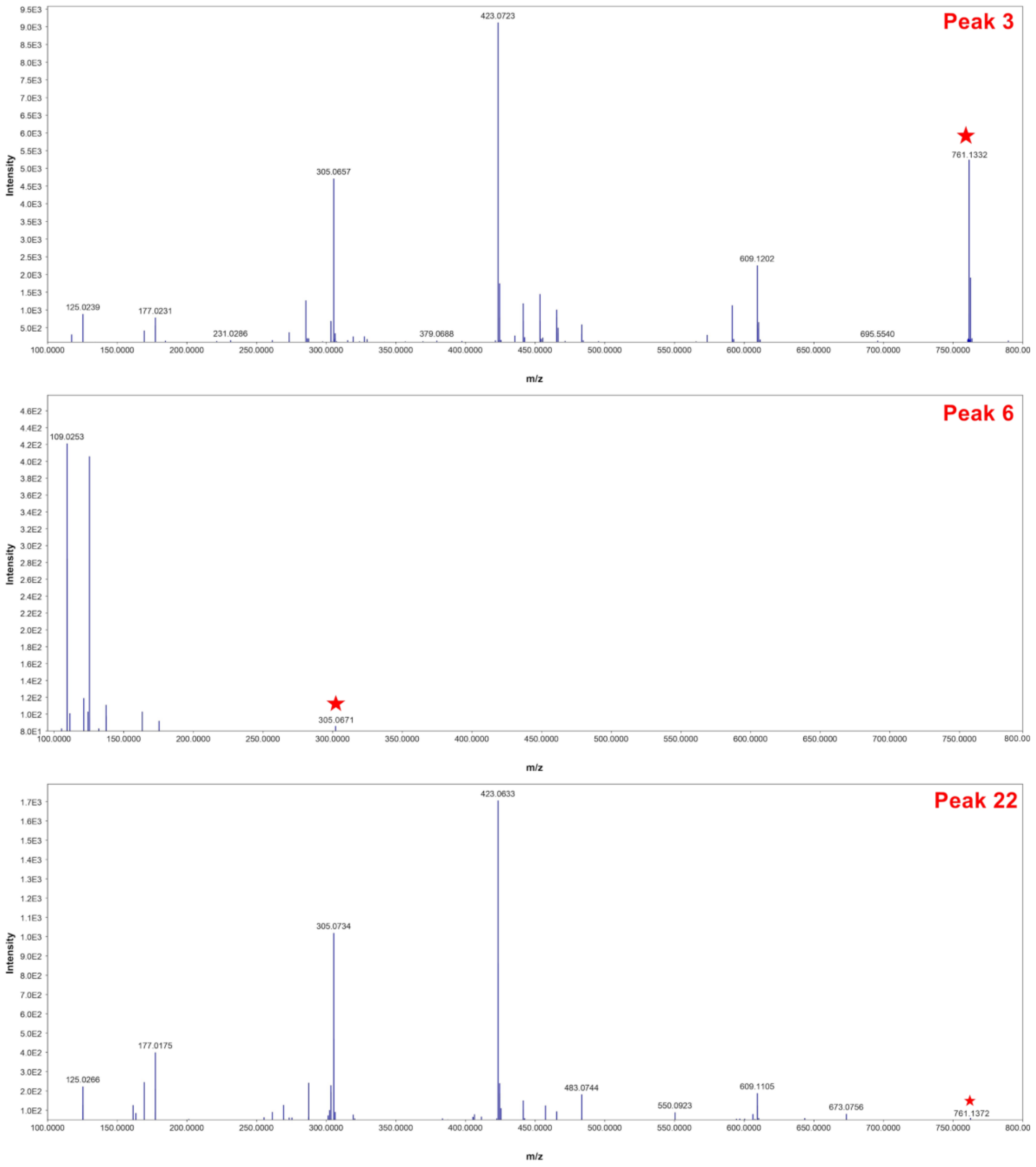

Figure. S2. Matches of MS/MS spectra for some compounds (**3**, **6**, **22**) flavan-3-ols derivatives (G2); Red star: parental ion

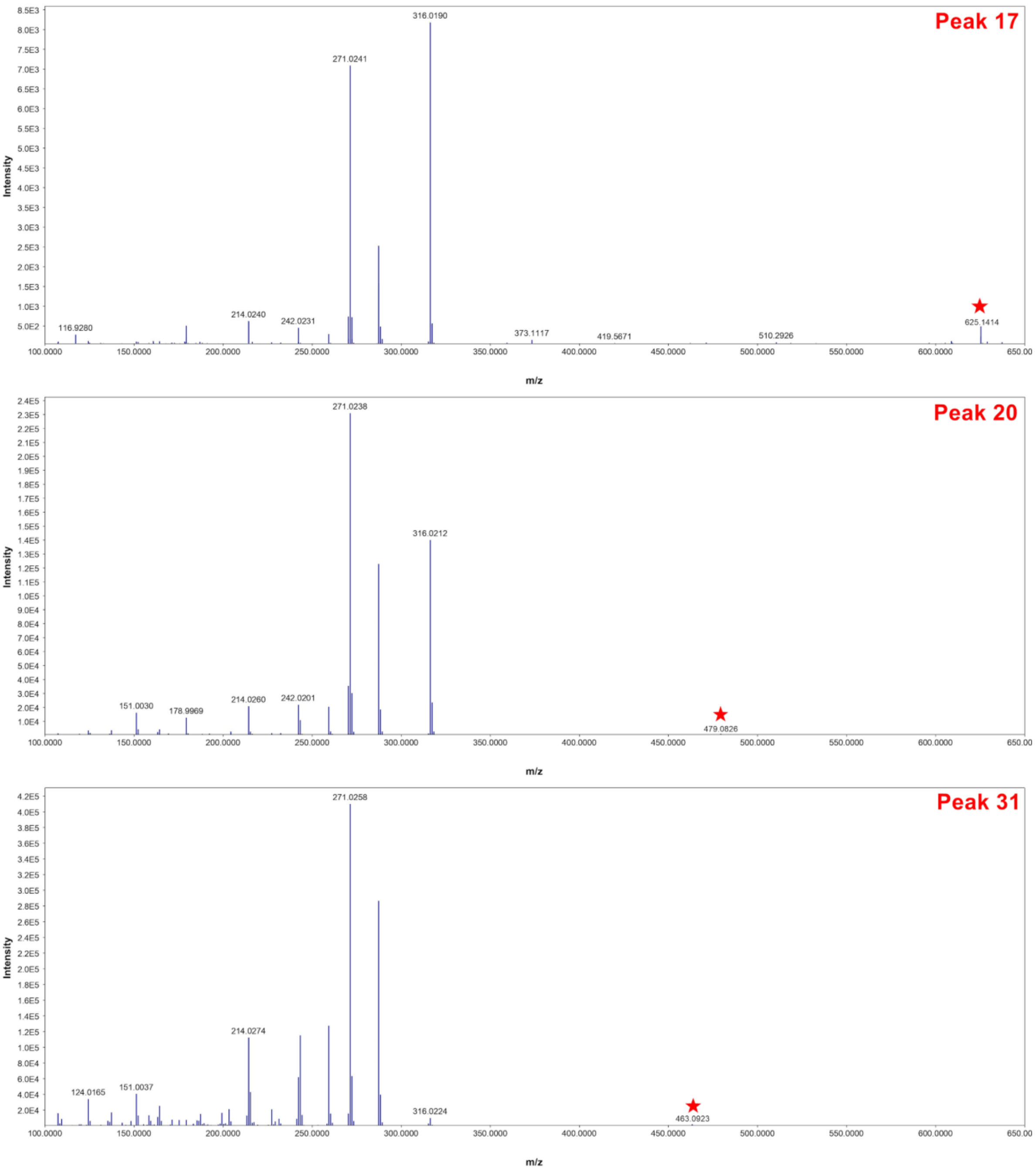

Figure. S3. Matches of MS/MS spectra for some compounds (**17**, **20**, **31**) flavones derivatives (G3); Red star: parental ion

Structural description of Compounds Annotated in GNPS

Compound **4** was assigned to gallocatechin, with yielded the fragment ions of *m/z* 261, 219, 179 and 125, as reported [1]. An isomer assigned to (epi)gallocatechin **(6)** was also detected and confirmed with the available experimental MS^2 spectrum in MoNA (Spectrum VF-NPL-QEHF013806). Both **4** and **6** had already been largely described in *Stryphnodendron adstringens, Stryphnodendron polyphyllum* and *Stryphnodendron rotundifolium* [2–11].

The $[M-H]^-$ of **9** (7.30 min) of *m/z* 483.0786 and of **10** (7.41 min) of *m/z* 483.0792, both with the same molecular formula $C_{20}H_{20}O_{14}$ have been assigned to [3,4,5-trihydroxy-6-(3,4,5-trihydroxybenzoyl)oxyoxan-2-yl]methyl-3,4,5-trihydroxybenzoate isomers. According to the spectral matching in the GPNS database, these molecules have two gallate units in positions 1 and 6 of the hexose. Both compounds presented identical fragmentation patterns (Figure 3A). The fragment ion of *m/z* 331 corresponds to the loss of one gallate unit $[M–H–C_7H_4O_4]^-$ whereas that of *m/z* 313 to dehydration of $[M–H–C_7H_4O_4–H_2O]^-$ of *m/z* 331. Loss of CO2 yields the fragment ion of *m/z* 287 $[M–H–C_7H_4O_4–CO_2]^-$. Subsequently CH_4 loss yields *m/z* 271 $[M–H–C_7H_4O_4–CO_2–CH_4]^-$. The ion of *m/z* 211 is likely formed by $C_2H_4O_2$ loss from the residual hexose $[M–H–272–C_2H_4O_2]^-$. Fragment ions of *m/z* 169 $[M–H–272–C_2H_4O_2–C_2H_2O]^-$ and *m/z* 125 $[M–H–272–C_2H_4O_2–C_2H_2O–CO_2]^-$ are produced likely as described for **1**.

Compounds **13**, **16** and **18** were assigned to (epi)gallocatechin gallate (EGCG), Taxifolin-4'-glucoside and gallocatechin gallate (GCG), respectively. They all yielded well known mass transitions (169→125) which confirmed a gallate group. For EGCG (**13**) and GCG (**18**), fragments have been well described, including their profiles and retention times in different chromatographic conditions [12,13]. Taxifolin-4'-glucoside yielded fragment ions of *m/z* 447 after H2O loss followed by hexose loss (*m/z* 285). The ion of *m/z* 421 resulted from CO_2 loss $[M–H–CO_2]^-$ [14] whereas *m/z* 357 from the partial loss of glycoside $[M–H–H_2O–C_3H_6O_3]^-$ ($^{0,3}X_1$) [15]. The fragment ions of *m/z* 313 ($^{1,3}B^-$) and *m/z* 151 ($^{1,3}A^-$) are likely fragments from RDA in ring C [14]. In particular, compound **13** is part of the cluster of gallic acid derivatives shown in Figure 1, this is probably due to the high intensity of the gallate fragment ($[M-H]^-$ at m/z 169).

Compounds **19** with $[M-H]^-$ of *m/z* 479.0824 (111.55 min) and **20** with $[M-H]^-$ of *m/z* 479.0826 (11.86 min) were assigned to myricetin 3-galactoside isomers and their set of fragment ions (*m/z* 316, 287, 271, 259, 243, 214, 151 and 124) were confirmed with the spectral matching in GNPS and the reported data [16]. Compound **17** with $[M-H]^-$ of *m/z* 625.1414 was also assigned via spectral matching against the experimental database as myricetin-3-*O*-hexosyl-deoxyhexoside.

Compounds **31**, **34**, **35**, **36** and **40** were isobaric and detected as $[M-H]^-$ of *m/z* 463 with a molecular formula of $C_{21}H_{20}O_{12}$. These were assigned via matching against experimental data on GNPS, to two myricitrin isomers (**31** and **35**), myricetin-3-*O*-rhamnopyranoside (**34**), isoquercitrin (**36**) and quercetin 4-glucoside (**40**).

Compounds **14** and **15** were detected at slightly different retention times, 9.77 min and 10.18 min, respectively. For a MF of $C_{20}H_{20}O_{13}$, they were assigned to 3,4,5-trihydroxybenzaldehyde-3-*O*-[6-*O*-(3,4,5-trihydroxybenzoyl)-glucopyranoside] isomers. Both isomers showed the same fragment ion of *m/z* 449 for dehydration $[M–H–H_2O]^-$, *m/z* 423 for decarboxylation $[M–H–CO_2]^-$, *m/z* 315 for loss of a gallate unit $[M–H–C_7H_4O_4]^-$, *m/z* 313 for loss of a gallic aldehyde $[M–H–C_7H_6O_4]^-$, *m/z* 193 for cleavage of the hexose moity ($^{1,3}X_1$) $[M–H–C_{11}H_{14}O_8]^-$, *m/z* 169 for the gallate unit, *m/z* 152 for loss of the sugar and gallate units $[M–H–C_{13}H_{15}O_9{}^\bullet]^{-\bullet}$ and *m/z* 125 for decarboxylation of a gallate unit $[M–H–298–CO_2]^-$.

Compound **21** was postulated as coumaroyl-*O*-galloyl-glucose after comparison with the reported MS^2 [17–19]. This molecular ion yielded fragment ions of *m/z* 316 ($[M–C_6H_{10}O_5]^-$), *m/z* 313 for loss of the coumaroyl group $[M–H–C_9H_8O_3]^-$, *m/z* 271 for $[M–H–C_7H_{10}O_7]^-$ and *m/z* 169 for the gallate unit. Compound **25** yielded a $[M-H]^-$ of *m/z* 495.1510 (12.86 min) whereas **26** yielded a $[M-H]^-$ of *m/z* 493.1359 (12.88 min), which were assigned to (3,4,5-trihydroxy-6-(4-(1-hydroxypropan-2-yl)-2-methoxyphenoxy)tetrahydro-2H-pyran-2-yl)methyl 3,4,5-trihydroxybenzoate, and (3,4,5-trihydroxy-6-(2-hydroxy-4-(3-oxobutyl)phenoxy)tetrahydro-2H-

pyran-2-yl)methyl 3,4,5-trihydroxybenzoate, respectively. These ions produced the same set of MS^2 ions of *m/z* 313, 181, 179, 169, 151 and 125, indicating a similar or common core structure. The mass difference for the first fragment ion of *m/z* 313 suggest neutral loss of 4-(1-hydroxypropan-2-yl)-2-methoxyphenol $[M–H–C_{10}H_{14}O_3]^-$ for **25** and 4-(3,4-dihydroxyphenyl)butan-2-one $[M–H–C_{10}H_{12}O_3]^-$ for **26**.

Compounds **32**, **38** (singleton in the MN), and **43** are isomers with MF $C_{23}H_{28}O_{11}$ and retention times of 13.56, 14.71 and 16.53 min, respectively. Based on the *in silico* database, they were assigned as 3,5-dihydroxy-2-(4-hydroxy-2-isopropyl-5-methylphenoxy)-6-(hydroxymethyl)tetrahydro-2H-pyran-4-yl-3,4,5-trihydroxybenzoate**,** 6''-*O*-galloylepirhododendrin, and querglanin. For **32** and **38**, the fragment ion of *m/z* 313 results from $C_{10}H_{14}O_2$ loss, pointing to a hexose linked to a gallate unit. The fragment ion of *m/z* 151 is likely formed from H_20 loss of the ion of *m/z* 169 followed by CO_2 loss that yields the ion of *m/z* 123. The fragment ion of *m/z* 165 likely results from $C_{13}H_{15}O_9^{\bullet}$ loss (galloyl-hexose moiety). Compound **44** (singleton in the MN) was assigned as phlorizin chalcone. It yielded fragment ions at *m/z* 313, 169, 151, 125 and 119. This compound seems to correspond to a monosaccharide of trihydroxylated chalcone (positions 4, 4', 6) attached to a hexose at position C2'. Cleavage of bond C8-C9 generates fragment ions of *m/z* 313 $[M–H–C_8H_8O]^-$ and *m/z* 119 $[M–H–C_{13}H_{14}O_9]^-$. For the fragment ions of *m/z* 169, 151, and 125, we observed the same products as previously discussed for **32** and **38**.

Compound **12** was identified as 3,5,7-trihydroxyflavanone-5-*O*-[galactopyranosyl-glucopyranoside]. The fragment ion of *m/z* 415 likely results from the loss of a glycoside unit $[M–H–C_6H_{12}O_6]^-$ followed by CH_2O loss to *m/z* 385 $[M–H–C_6H_{12}O_6–CH_2O]^-$ [20]. A fragment ion of *m/z* 355 is likely formed by $^{1,3}X_1$ cleavage $[M–H–C_2H_4O_2]^-$. Similarly, the ion of *m/z* 343 results from *m/z* 415 via $^{0,3}X_1$ fragmentation $[M–H–C_3H_4O_2]^-$, followed by H2O loss that yields *m/z* 325 $[M–H–C_2H_4O_2–H_2O]^-$. An heterocyclic cleavage and loss of the glucoside residue likely yields *m/z* 235 $[M–H–180–C_3H_6O_3]^-$. Fragment ion of *m/z* 313 $[M–H–C_4H_6O_3]^-$ likely results from *m/z* 415 by $^{0,2}X_1$ fragmentation. The ion of *m/z* 193 is likely from RDA at ring C from *m/z* 415 $[M–H–C_4H_6O_3–C_8H_8O]^-$. The aglycone is detected as the ion of *m/z* 271 $[M–H–C_{12}H_{20}O_{10}]^-$ after loss of two glucoside units. The ion of *m/z* 119 is likely a fragment from the aglycone after RDA at ring C $[M–H–2Glc–C_7H_4O_4]^-$.

Compound **16** was assigned as taxifolin-4'-glucoside. It yielded fragment ions of *m/z* 447 after H2O loss followed by hexose loss (*m/z* 285). The ion of *m/z* 421 resulted from CO_2 loss $[M–H–CO_2]^-$ [14] whereas *m/z* 357 from the partial loss of glycoside $[M–H–H_2O–C_3H_6O_3]^-$ ($^{0,3}X_1$) [15]. The fragment ions of *m/z* 313 ($^{1,3}B^-$) and *m/z* 151 ($^{1,3}A^-$) are likely fragments from RDA in ring C [14]. Compound **24** with $[M – H]^-$ of *m/z* 471.0918 was assigned to 3-*O*-galloyl-4'-*O*-methylepigallocatechin already reported in *S. adstringens* [9] and its fragmentation pattern matched that of the authentic standard [21].

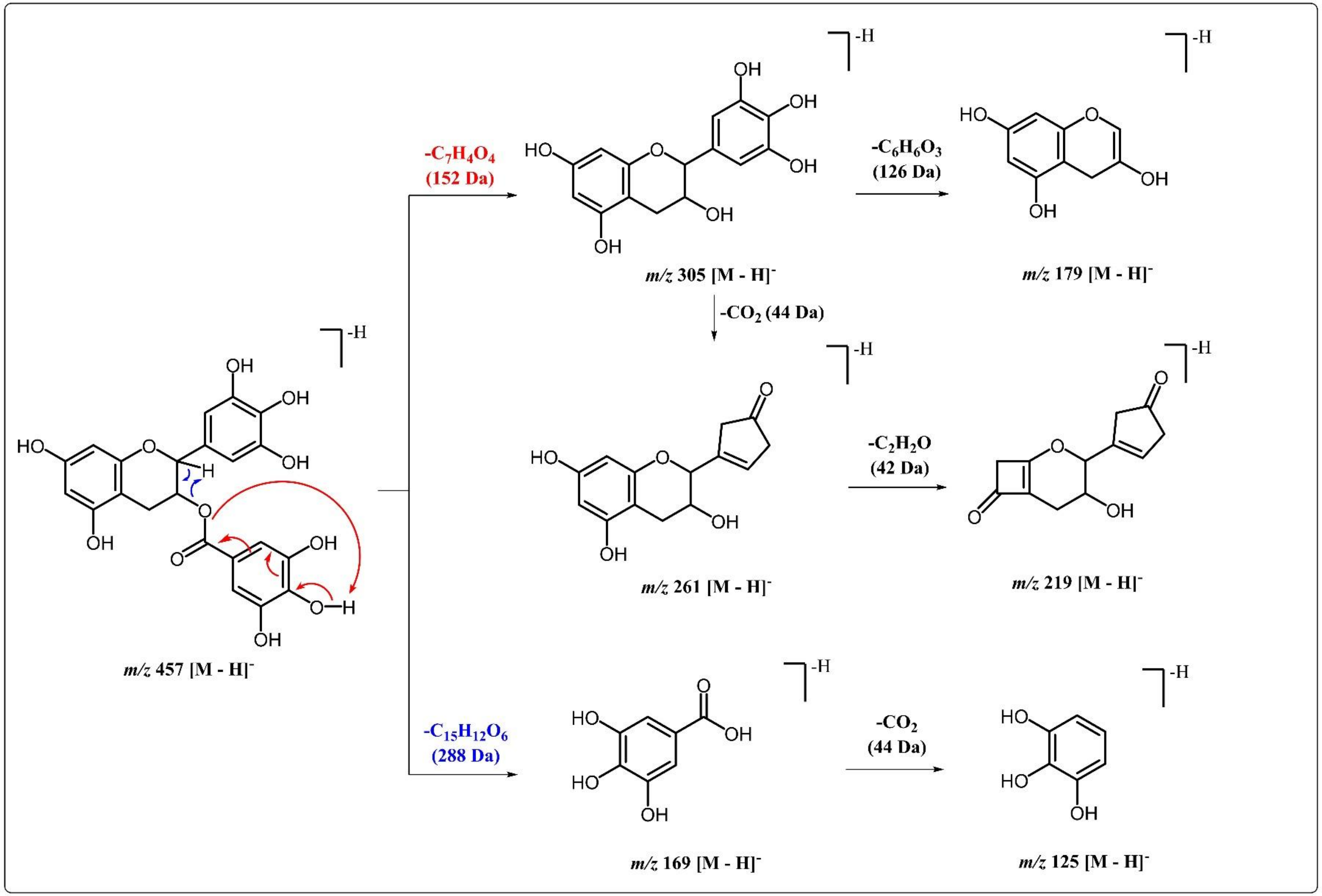

Figure. S4. Proposed fragmentation scheme of deprotonated (epi)gallocatechin gallate (**13**). Note: mechanisms based on the literature [1,29]**.**

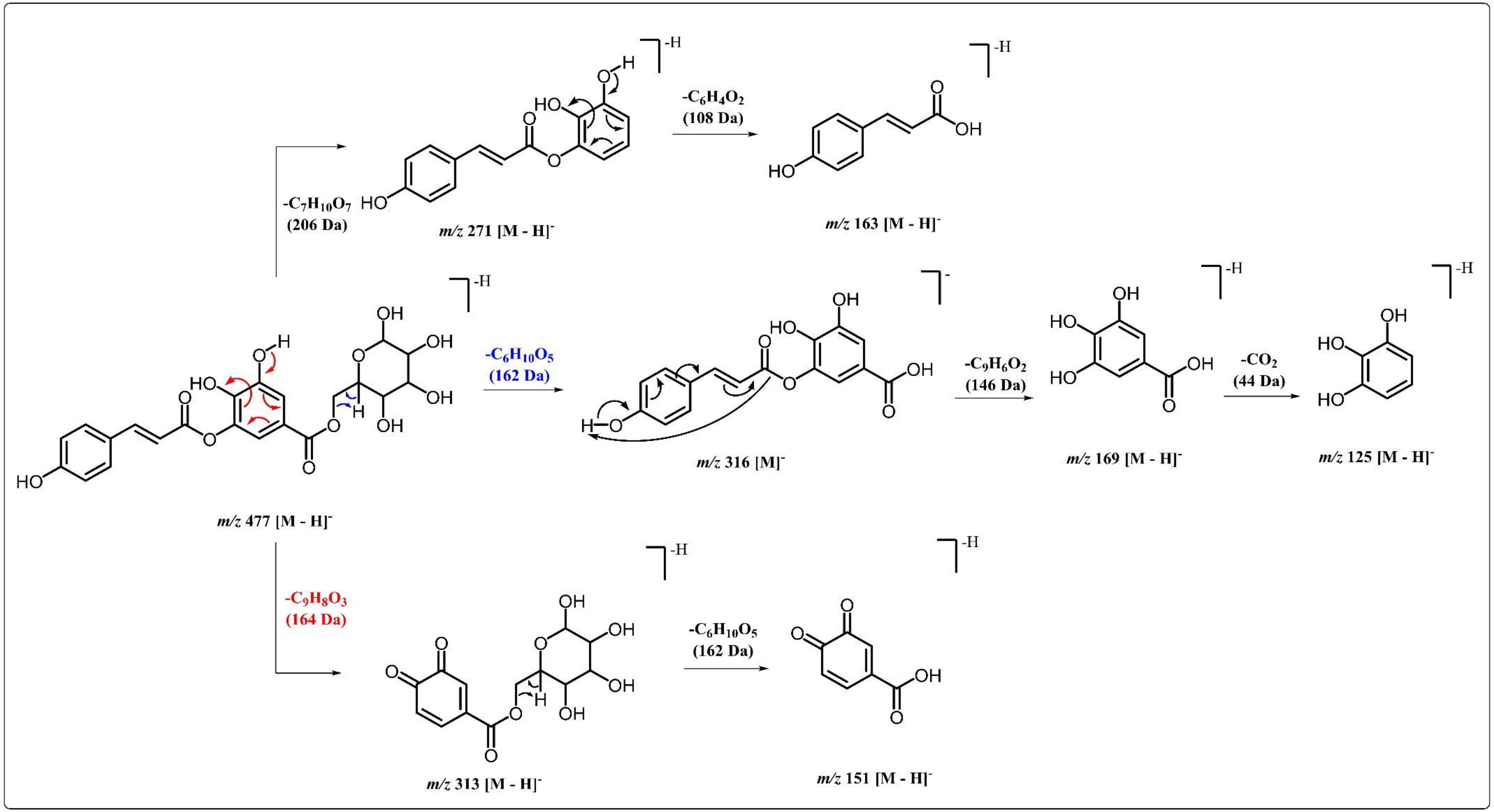

Figure. S5. Proposed fragmentation scheme of deprotonated *O*-coumaroyl *O*-galloyl-hexoside (**21**). Note: mechanisms based on the literature [17–19]**.**

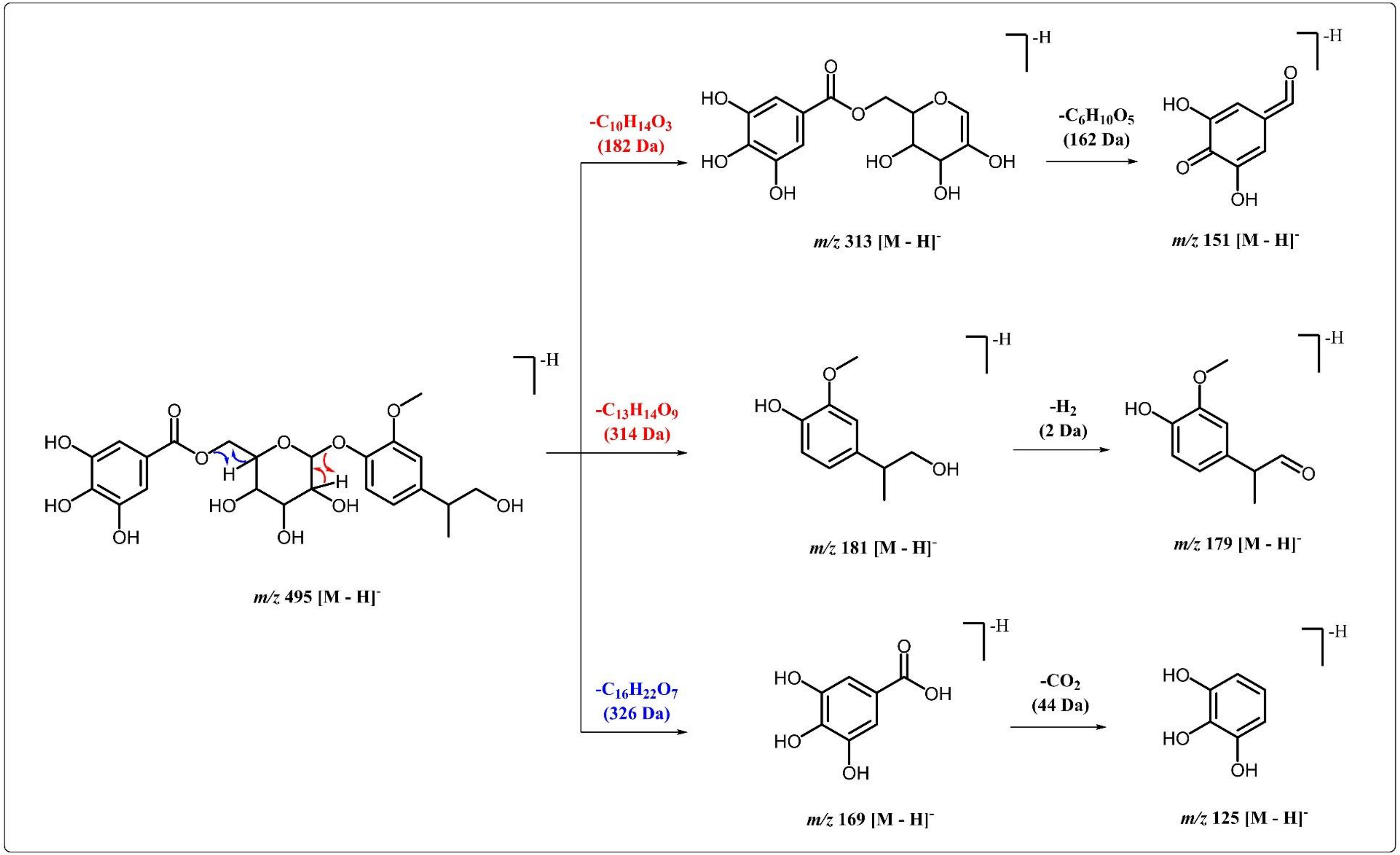

Figure. S6. Proposed fragmentation scheme of deprotonated (3,4,5-trihydroxy-6-(4-(1-hydroxypropan-2-yl)-2-methoxyphenoxy)tetrahydro-2H-pyran-2-yl)methyl 3,4,5-trihydroxybenzoate (**25**). Note: mechanisms based on the literature [30,31]**.**

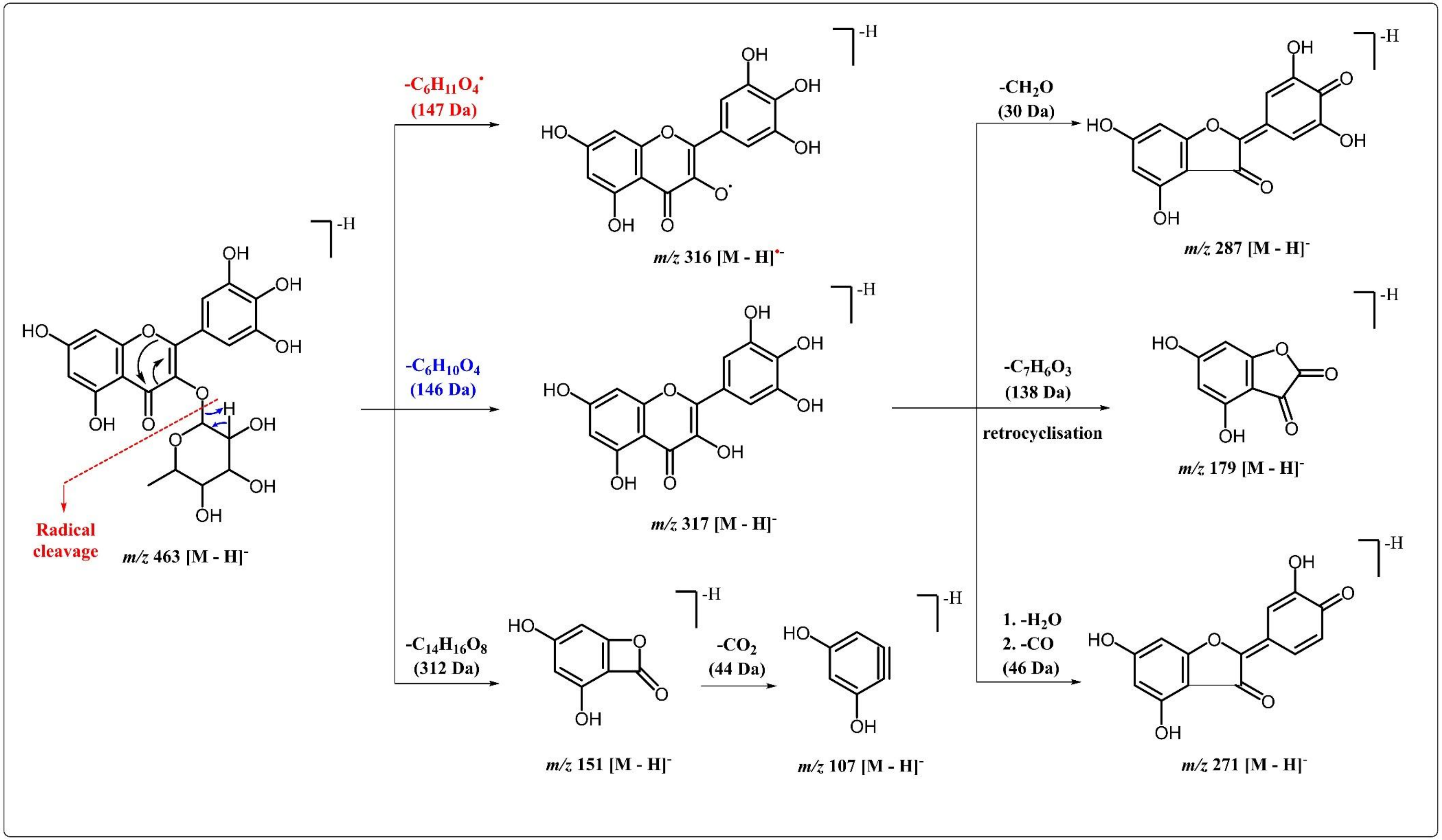

Figure. S7. Proposed fragmentation scheme of deprotonated myricitrin (**31**). Note: mechanisms based on the literature [14,32–34].

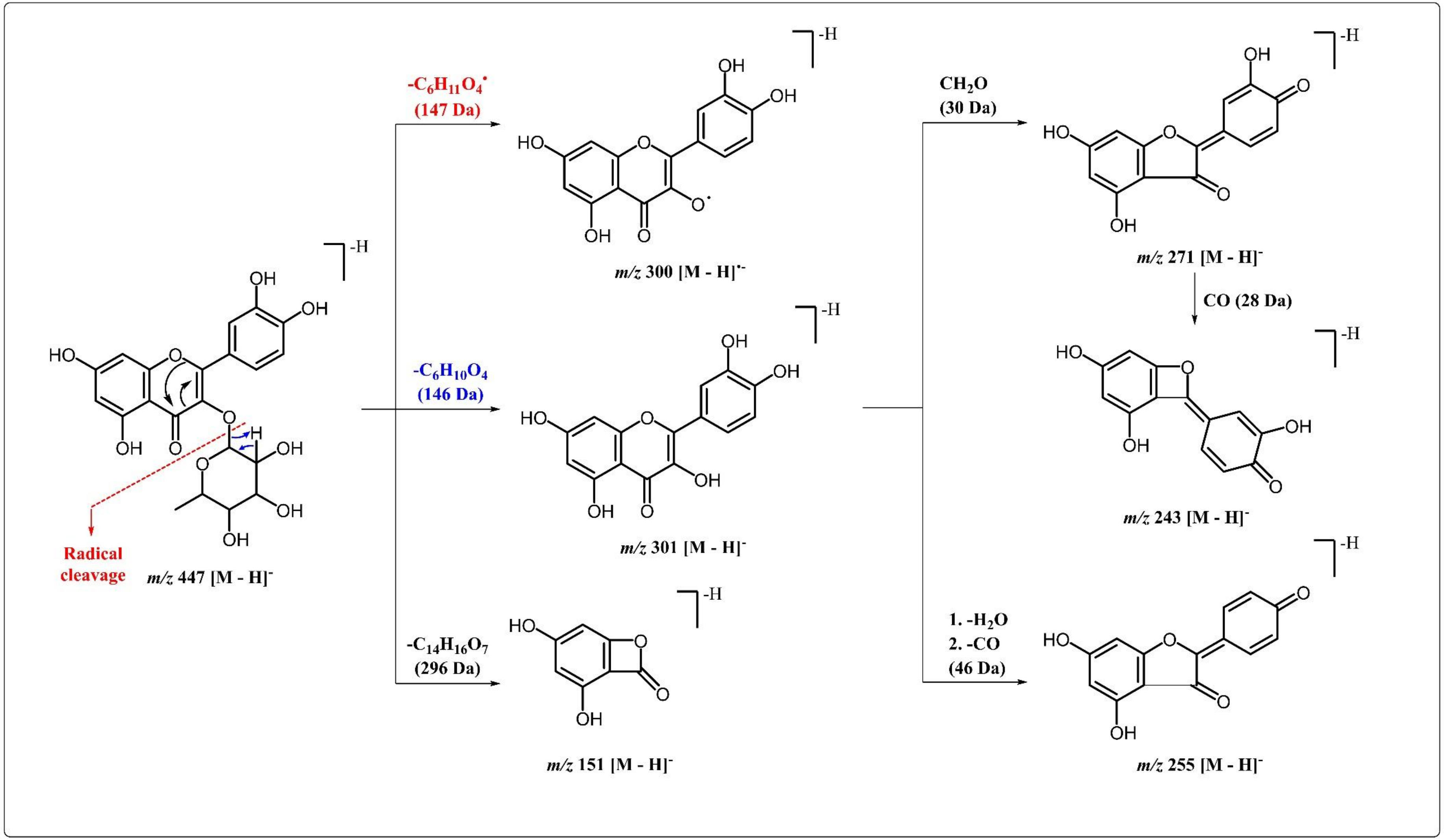

Figure. S8. Proposed fragmentation scheme of deprotonated quercitrin (**41**). Note: mechanisms based on the literature [14,32–34].

Reference

1. Zheng, Y.; Zeng, X.; Chen, T.; Peng, W.; Su, W. Chemical Profile, Antioxidative, and Gut Microbiota Modulatory Properties of Ganpu Tea: A Derivative of Pu-erh Tea. *Nutrients* **2020**, *12*, doi:10.3390/nu12010224.
2. Lopes, G.C.; Vieira Machado, F.A.; Mendes de Toledo, C.E.; Sakuragui, C.M.; Palazzo de Mello, J.C. Chemotaxonomic significance of 5-deoxyproanthocyanidins in Stryphnodendron species. *Biochem. Syst. Ecol.* **2008**, *36*, 925–931, doi:10.1016/j.bse.2008.10.004.
3. Nascimento, A.M. do; Guedes, P.T.; Castilho, R.O.; Vianna-Soares, C.D. Stryphnodendron adstringens (Mart.) Coville (Fabaceae) proanthocyanidins quantitation by RP-HPLC. *Braz. J. Pharm. Sci.* **2013**, *49*, 549–558, doi:10.1590/S1984-82502013000300016.
4. Santos, S.C.; Costa, W.F.; Ribeiro, J.P.; Guimarães, D.O.; Ferri, P.H.; Ferreira, H.D.; Seraphin, J.C. Tannin composition of barbatimão species. *Fitoterapia* **2002**, *73*, 292–299, doi:10.1016/s0367-326x(02)00081-3.
5. Henriques, B.O.; Corrêa, O.; Azevedo, E.P.C.; Pádua, R.M.; Oliveira, V.L.S. de; Oliveira, T.H.C.; Boff, D.; Dias, A.C.F.; Souza, D.G. de; Amaral, F.A.; et al. In Vitro TNF- Inhibitory Activity of Brazilian Plants and Anti-Inflammatory Effect of Stryphnodendron adstringens in an Acute Arthritis Model. *Evid. Based. Complement. Alternat. Med.* **2016**, *2016*, doi:10.1155/2016/9872598.
6. Ribeiro, T.G.; Nascimento, A.M.; Henriques, B.O.; Chávez-Fumagalli, M.A.; Franca, J.R.; Duarte, M.C.; Lage, P.S.; Andrade, P.H.R.; Lage, D.P.; Rodrigues, L.B.; et al. Antileishmanial activity of standardized fractions of Stryphnodendron obovatum (Barbatimão) extract and constituent compounds. *J. Ethnopharmacol.* **2015**, *165*, 238–242, doi:10.1016/j.jep.2015.02.047.
7. Pinto, S.C.G.; Bueno, F.G.; Panizzon, G.P.; Morais, G.; Dos Santos, P.V.P.; Baesso, M.L.; Leite-Mello, E.V. de S.; de Mello, J.C.P. Stryphnodendron adstringens: Clarifying Wound Healing in Streptozotocin-Induced Diabetic Rats. *Planta Med.* **2015**, *81*, 1090–1096, doi:10.1055/s-0035-1546209.
8. Felipe, A.M.M.; Rincão, V.P.; Benati, F.J.; Linhares, R.E.C.; Galina, K.J.; de Toledo, C.E.M.; Lopes, G.C.; de Mello, J.C.P.; Nozawa, C. Antiviral effect of Guazuma ulmifolia and Stryphnodendron adstringens on poliovirus and bovine herpesvirus. *Biol. Pharm. Bull.* **2006**, *29*, 1092–1095, doi:10.1248/bpb.29.1092.
9. de Mello, J.P.; Petereit, F.; Nahrstedt, A. Flavan-3-ols and prodelphinidins from Stryphnodendron adstringens. *Phytochemistry* **1996**, *41*, 807–813, doi:10.1016/0031-9422(95)00686-9.
10. Trevisan, D.A.C.; da Silva, P.V.; Farias, A.B.P.; Campanerut-Sá, P.A.Z.; Ribeiro, T.D.V.R.; Faria, D.R.; de Mendonça, P.S.B.; de Mello, J.C.P.; Seixas, F.A.V.; Mikcha, J.M.G. Antibacterial activity of Barbatimão (Stryphnodendron adstringens) against Staphylococcus aureus: in vitro and in silico studies. *Lett. Appl. Microbiol.* **2020**, doi:10.1111/lam.13317.
11. Giffoni de Carvalho, J.T.; Henao Agudelo, J.S.; Baldivia, D.D.S.; Carollo, C.A.; Silva, D.B.; de Picoli Souza, K.; Saraiva Câmara, N.O.; Dos Santos, E.L. Hydroethanolic stem bark extracts of Stryphnodendron adstringens impair M1 macrophages and promote M2 polarization. *J. Ethnopharmacol.* **2020**, *254*, 112684, doi:10.1016/j.jep.2020.112684.
12. Yuzuak, S.; Ballington, J.; Xie, D.-Y. HPLC-qTOF-MS/MS-Based Profiling of Flavan-3-ols and Dimeric Proanthocyanidins in Berries of Two Muscadine Grape Hybrids FLH 13-11 and FLH 17-66. *Metabolites* **2018**, *8*, doi:10.3390/metabo8040057.
13. Spáčil, Z.; Nováková, L.; Solich, P. Comparison of positive and negative ion detection of tea catechins using tandem mass spectrometry and ultra high performance liquid chromatography. *Food Chem.* **2010**, *123*, 535–541, doi:10.1016/j.foodchem.2010.04.048.
14. Fabre, N.; Rustan, I.; de Hoffmann, E.; Quetin-Leclercq, J. Determination of flavone, flavonol, and flavanone aglycones by negative ion liquid chromatography electrospray ion trap mass spectrometry. *J. Am. Soc. Mass Spectrom.* **2001**, *12*, 707–715, doi:10.1016/S1044-0305(01)00226-4.
15. Roowi, S.; Crozier, A. Flavonoids in tropical citrus species. *J. Agric. Food Chem.* **2011**, *59*, 12217–12225, doi:10.1021/jf203022f.
16. Singh, A.P.; Wilson, T.; Luthria, D.; Freeman, M.R.; Scott, R.M.; Bilenker, D.; Shah, S.; Somasundaram, S.; Vorsa, N. LC-MS–MS characterisation of curry leaf flavonols and antioxidant activity. *Food Chem.* **2011**, *127*, 80–85, doi:10.1016/j.foodchem.2010.12.091.
17. Hofmann, T.; Nebehaj, E.; Albert, L. Antioxidant properties and detailed polyphenol profiling of European hornbeam (Carpinus betulus L.) leaves by multiple antioxidant capacity assays and high-performance liquid chromatography/multistage electrospray mass spectrometry. *Industrial Crops and Products* 2016, *87*, 340–349.
18. Wang, J.-B.; Qin, Y.; Kong, W.-J.; Wang, Z.-W.; Zeng, L.-N.; Fang, F.; Jin, C.; Zhao, Y.-L.; Xiao, X.-H. Identification of the antidiarrhoeal components in official rhubarb using liquid chromatography–

tandem mass spectrometry. *Food Chem.* **2011**, *129*, 1737–1743, doi:10.1016/j.foodchem.2011.06.041.

19. Santos, H.F.D.; Campos, J.F.; Santos, C.M.D.; Balestieri, J.B.P.; Silva, D.B.; Carollo, C.A.; de Picoli Souza, K.; Estevinho, L.M.; Dos Santos, E.L. Chemical Profile and Antioxidant, Anti-Inflammatory, Antimutagenic and Antimicrobial Activities of Geopropolis from the Stingless Bee Melipona orbignyi. *Int. J. Mol. Sci.* **2017**, *18*, doi:10.3390/ijms18050953.
20. Morreel, K.; Kim, H.; Lu, F.; Dima, O.; Akiyama, T.; Vanholme, R.; Niculaes, C.; Goeminne, G.; Inzé, D.; Messens, E.; et al. Mass spectrometry-based fragmentation as an identification tool in lignomics. *Anal. Chem.* **2010**, *82*, 8095–8105, doi:10.1021/ac100968g.
21. Chen, S.; Lin, J.; Liu, H.; Gong, Z.; Wang, X.; Li, M.; Aharoni, A.; Yang, Z.; Yu, X. Insights into Tissue-specific Specialized Metabolism in Tieguanyin Tea Cultivar by Untargeted Metabolomics. *Molecules* **2018**, *23*, doi:10.3390/molecules23071817.
22. Joseph, R.C.; Silva da Fonseca Diniz, M.; Magno do Nascimento, V.; Barbosa Muribeca, A. de J.; Costa Santiago, J.C.; da Cunha Borges, L.; da Costa Sá, P.R.; Portal Gomes, P.W.; da Silva Cardoso, J.C.; Rocha de Castro, M.N.; et al. Secure and Sustainable Sourcing of Plant Tissues for the Exhaustive Exploration of Their Chemodiversity. *Molecules* **2020**, *25*, doi:10.3390/molecules25245992.
23. da Costa, J.G.M.; Leite, G. de O.; Dubois, A.F.; Seeger, R.L.; Boligon, A.A.; Athayde, M.L.; Campos, A.R.; da Rocha, J.B.T. Antioxidant effect of Stryphnodendron rotundifolium Martius extracts from Cariri-Ceará State (Brazil): potential involvement in its therapeutic use. *Molecules* **2012**, *17*, 934–950, doi:10.3390/molecules17010934.
24. Palazzo de Mello, J.; Petereit, F.; Nahrstedt, A. Prorobinetinidins from Stryphnodendron adstringens. *Phytochemistry* **1996**, *42*, 857–862, doi:10.1016/0031-9422(95)00953-1.
25. Oliveira, D.A.J.; Amaral, J.G.; Garcia, L.B.; Dos Santos, M.S.; Silva, L.A.O.; Almeida, M.P.; Gomes, A.F.; Barros, D.R.P.; Lopes, N.P.; Pereira, G.R.; et al. Associating chitosan and microemulsion as a topical vehicle for the administration of herbal medicines. *Carbohydr. Polym.* **2021**, *255*, 117482, doi:10.1016/j.carbpol.2020.117482.
26. Sousa, J.N.; Pedroso, N.B.; Borges, L.L.; Oliveira, G.A.R.; Paula, J.R.; Conceição, E.C. Optimization of Ultrasound-assisted extraction of polyphenols, tannins and epigallocatechin gallate from barks of Stryphnodendron adstringens (Mart.) Coville bark extracts. *Pharmacogn. Mag.* **2014**, *10*, S318–23, doi:10.4103/0973-1296.133287.
27. Palazzo de Mello, J.C.; Petereit, F.; Nahrstedt, A. A dimeric proanthocyanidin from Stryphnodendron adstringens. *Phytochemistry* **1999**, *51*, 1105–1107, doi:10.1016/S0031-9422(98)00715-8.
28. Ishida, K.; de Mello, J.C.P.; Cortez, D.A.G.; Filho, B.P.D.; Ueda-Nakamura, T.; Nakamura, C.V. Influence of tannins from Stryphnodendron adstringens on growth and virulence factors of Candida albicans. *J. Antimicrob. Chemother.* **2006**, *58*, 942–949, doi:10.1093/jac/dkl377.
29. Castro, C.; Luz, L.; Guedes, J.; Porto, D.; Silva, M.F.; Silva, G.; Ribeiro, P.; Canuto, K.; Brito, E.; Zampieri, D.; et al. Metabolomics-Based Discovery of Biomarkers with Cytotoxic Potential in Extracts of Myracrodruon urundeuva. *J. Braz. Chem. Soc.* **2020**, doi:10.21577/0103-5053.20190242.
30. Demarque, D.P.; Crotti, A.E.M.; Vessecchi, R.; Lopes, J.L.C.; Lopes, N.P. Fragmentation reactions using electrospray ionization mass spectrometry: an important tool for the structural elucidation and characterization of synthetic and natural products. *Nat. Prod. Rep.* **2016**, *33*, 432–455, doi:10.1039/c5np00073d.
31. Tsugawa, H.; Kind, T.; Nakabayashi, R.; Yukihira, D.; Tanaka, W.; Cajka, T.; Saito, K.; Fiehn, O.; Arita, M. Hydrogen Rearrangement Rules: Computational MS/MS Fragmentation and Structure Elucidation Using MS-FINDER Software. *Anal. Chem.* **2016**, *88*, 7946–7958, doi:10.1021/acs.analchem.6b00770.
32. Tiberti, L.A.; Yariwake, J.H.; Ndjoko, K.; Hostettmann, K. On-line LC/UV/MS analysis of flavonols in the three apple varieties most widely cultivated in Brazil. *J. Braz. Chem. Soc.* **2007**, *18*, 100–105, doi:10.1590/S0103-50532007000100011.
33. Li, A.; Hou, X.; Wei, Y. Fast screening of flavonoids from switchgrass and Mikania micrantha by liquid chromatography hybrid-ion trap time-of-flight mass spectrometry. *Anal. Methods* **2018**, *10*, 109–122, doi:10.1039/C7AY02103H.
34. Candela, L.; Formato, M.; Crescente, G.; Piccolella, S.; Pacifico, S. Coumaroyl Flavonol Glycosides and More in Marketed Green Teas: An Intrinsic Value beyond Much-Lauded Catechins. *Molecules* **2020**, *25*, doi:10.3390/molecules25081765.
